# Thermally Controlled Chiral Supramolecular Polymorphism in Water

**DOI:** 10.1002/anie.1190401

**Published:** 2026-05-02

**Authors:** Zulema Fernández, Yongsheng Li, Daniel Martínez, Julia Terlau, Myongsoo Lee, Bartolomé Soberats, Gustavo Fernández

**Affiliations:** ^1^ Organisch‐Chemisches Institut Universität Münster Münster Germany; ^2^ Department of Chemistry State Key Lab of Molecular Engineering of Polymers and Shanghai Key Lab of Molecular Catalysis and Innovative Materials, Fudan University Shanghai China; ^3^ Department of Chemistry Universitat de Les Illes Balears Palma de Mallorca Spain

**Keywords:** π‐conjugated systems, chirality, LCST, supramolecular polymerization, supramolecular polymorphism

## Abstract

Biological assemblies such as proteins adapt their helical morphology and function in response to external stimuli, yet controlled polymorphic transitions in synthetic chiral supramolecular analogues remain poorly understood. Herein, we demonstrate a strategy to achieve controlled chiral supramolecular polymorphism in water by coupling molecular design with external stimuli. An unsymmetrical oligo(phenyleneethynylene) derivative **1** bearing a pyridine unit, a hydrogen‐bonding amide group, and chiral hydrophilic side chains self‐assembles into three distinct chiral supramolecular polymorphs in water that are stable at different temperature regimes. At room temperature (RT), **1** self‐assembles into short cylinders (**AggI**), which undergo a polymorphic transition to transient double helical fibers upon heating around the LCST (**AggII**, ≈ 325 K) and ultimately to irregular planar aggregates (**AggIII**) above the LCST. Remarkably, the polymorphic transitions are linked to the temperature‐dependent conformation and degree of dehydration of the glycol chains. Although **AggII** exists only within a narrow temperature window in pristine water, it can be stabilized and isolated at RT through chemical stimuli such as co‐solvents or metal salts that modulate the LCST. Our results establish LCST‐coupled chirality as a powerful strategy to regulate thermoresponsive supramolecular polymorphism and offer potential strategies for the design of adaptive materials.

## Graphical abstract

Using an amphiphilic oligo(phenyleneethynylene) derivative, we establish LCST‐coupled chirality as a strategy to regulate thermoresponsive supramolecular polymorphism in water. At room temperature, **AggI** forms discrete assemblies that transform into elongated helical fibers (**AggII**) near the LCST and subsequently to sheet‐like structures (**AggIII**) at higher temperatures, yielding three stable polymorphs in distinct temperature regimes.

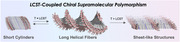

Biological entities such as proteins self‐assemble into distinct supramolecular architectures either through chemical modification of the monomer units or, more commonly, by adopting different packing morphologies while retaining the same chemical structure, a phenomenon known as polymorphism [1, 2, 3]. A particularly illustrative example is the tobacco mosaic virus, whose capsid proteins undergo morphological transitions from rod‐shaped helical particles to fibers or aggregates with distinct packing arrangements in response to external stimuli such as changes in pH or ionic strength [4]. This structural diversity has inspired the development of synthetic systems that mimic such behavior, enabling the creation of stimuli‐responsive and adaptive materials with potential applications across biology, materials science, and chemistry [5, 6, 7, 8].

In chemistry, polymorphism has long been a central concept in crystallography [6], yet its manifestation in solution‐phase supramolecular systems has only recently been recognized, a phenomenon termed supramolecular polymorphism [9, 10, 11]. Supramolecular polymorphs are particularly attractive because distinct and stable assembled states can be accessed from a single molecular building block in the same solvent, thereby encoding structural and functional diversity at the supramolecular level. Despite these appealing features, the rational design of systems that fulfill these criteria is challenging, which explains why examples of supramolecular polymorphs remain scarce [9, 10, 11, 12, 13, 14, 15, 16, 17, 18, 19, 20, 21, 22, 23]. This challenge is further accentuated in chiral supramolecular systems, where both equilibrium polymorphs and their controlled interconversion in water remain poorly understood. Such control is highly relevant for understanding supramolecular transitions in helical natural assemblies. Developing synthetic systems that enable controlled chiral supramolecular polymorphism in water thus represents an important step toward advancing adaptive materials and deepening our understanding of structure‐assembly relationships in biologically relevant environments.

Herein, we demonstrate controlled chiral supramolecular polymorphism in water by coupling molecular design and external stimuli. To this end, we designed an unsymmetrical oligo(phenyleneethynylene) (OPE) [24, 25, 26, 27, 28, 29, 30] derivative (**1**) bearing a terminal pyridine moiety, an amide group as a hydrogen bonding unit, and (*S*)‐chiral hydrophilic tetraethylene glycol side chains (see Supporting Information (S.I.) for synthesis and characterization). The glycol side chains not only impart water solubility but also efficiently transfer chiral information to the supramolecular stacks upon self‐assembly [31, 32, 33]. At room temperature (RT), compound **1** self‐assembles in water into a chiral supramolecular polymorph with a discrete size (**AggI**). Upon heating, conformational changes and progressive dehydration of the glycol chains give rise to a lower critical solution temperature (LCST), with an onset at approximately 327 K. Notably, slightly below this temperature (ca. 325 K), **AggI** transforms into a second chiral supramolecular polymorph with helical morphology (**AggII**), which subsequently evolves into a third polymorph composed of sheet‐like structures (**AggIII**) upon heating above the LCST (Scheme 1). All three states remain invariant over time at their respective temperature windows, confirming their polymorphic nature. Importantly, narrow‐window polymorph **AggII** can be stabilized and isolated at RT by chemical stimuli such as acetonitrile as a co‐solvent or metal salts, which lower the LCST. These results establish LCST‐coupled chirality as a powerful design principle to access and control multiple supramolecular polymorphs in aqueous environments.

**SCHEME 1:**
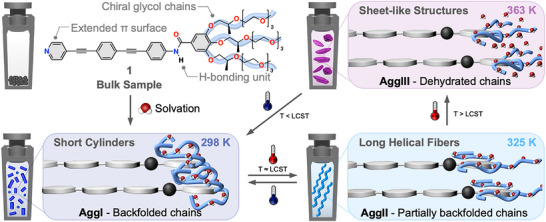
Molecular structure of **1** and schematic representation of its supramolecular polymorphism in water controlled by LCST.

To assess the self‐assembly behavior of **1**, we first performed solvent‐dependent spectroscopic studies using UV/Vis, Circular Dichroism (CD), and fluorescence spectroscopy. UV/Vis studies reveal that **1** remains molecularly dissolved in common organic solvents, displaying an absorption maximum at ca. *λ*_max_  =  337 nm (Figure 1a). In contrast, in water, a slight red‐shift of the absorption maximum (∆*λ*_max_ = 3 nm) is observed, accompanied by hypochromism, band sharpening, and the emergence of a low‐energy tail extending to ca. 450 nm (Figures 1a and S7). These spectral changes are indicative of aggregate formation (**AggI**), as further supported by CD and fluorescence spectroscopy (Figure 1b,c). Whereas CD spectra are silent in organic solvents, a negative Cotton band centered at ca. 360 nm appears in water, evidencing the transfer of chiral information from the stereogenic hydrophilic chains to the supramolecular stacks of **1** (Figure 1b). To understand this behavior, we calculated the CD and UV/Vis spectra of a previously optimized tetramer stack. Although the calculations predict a second Cotton effect, it is not experimentally resolved due to its low intensity and partial overlap with the dominant negative band, consistent with weak excitonic coupling and conformational flexibility of the glycol chains. Additionally, the pronounced red‐shift and enhanced emission observed in water compared to organic solvents suggest the formation of *J*‐type aggregates [34] displaying aggregation‐induced emission enhancement (AIEE) behavior (Figure 1c). Overall, the spectroscopic signatures of **AggI** closely reproduce the trends previously reported for a structurally related achiral analogue [35] and indicate a parallel arrangement of the monomer units with a small translational offset (Scheme 1), as corroborated by 2D NMR studies (Figures S8 and S9) and theoretical calculations (Figure S7g,h).

**FIGURE 1:**
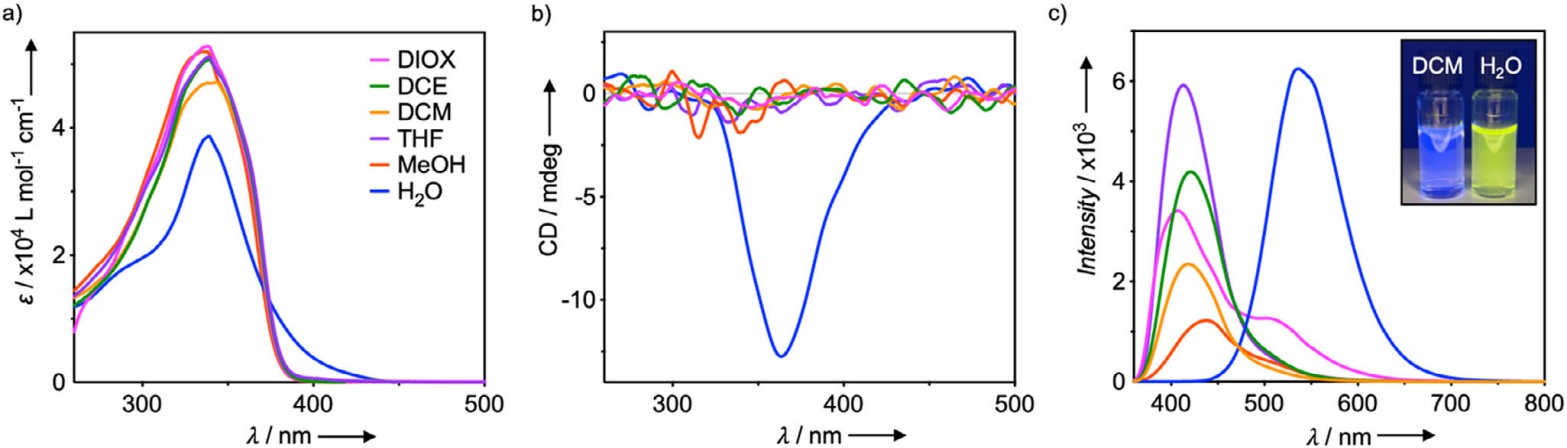
UV/Vis (a), CD (b), and emission (c) studies of **1** (298 K, *c*  =  20 µM, *λ*_exc_  =  340 nm) in different solvents (dioxane, 1,2‐dichloroethane, dichloromethane, tetrahydrofuran, methanol, and water). Inset in (c): solutions of **1** in dichloromethane and H_2_O under a 366 nm UV lamp illustrating the differences in emission.

Variable‐temperature (VT)‐UV/Vis studies were performed to gain insights into the self‐assembly mechanism of **1** (Figures 2a and S10). Notably, heating an aqueous solution of **AggI** from 298 to 363 K (1 K min^−1^) does not result in disassembly into the monomer species [36]. Instead, the absorption maximum at *λ*_max_ = 340 nm decreases markedly, accompanied by a pronounced increase in optical density at longer wavelengths (*T* > 325 K; *λ*_O.D._  =  500 nm), which is apparent from the increasing turbidity of the solution (Figure 2c, inset). Such behavior is characteristic of compounds bearing glycol chains, where heating induces dehydration and conformational rearrangement of these groups, ultimately resulting in macroscopic phase separation [37, 38, 39, 40, 41, 42, 43, 44, 45, 46, 47, 48, 49]. The temperature at which this process occurs is referred to as LCST, marking the loss of miscibility between solute and solvent. Closer inspection of the VT‐UV/Vis spectra below the LCST (*T*  =  327 K) reveals an initial hypsochromic shift of the absorption maximum (Δ*λ* = 3 nm), followed by a bathochromic shift of identical magnitude (Figure 2a). The latter effect, together with the reduced absorbance compared to **AggI**, indicates tighter chromophore packing and suggests the formation of a distinct aggregated species (**AggII**) (Figure 2a, light blue spectrum and Figure S11). This assignment is confirmed by VT‐CD studies (Figure 2b). Upon heating, the negative Cotton band of **AggI** at 360 nm progressively decreases and undergoes a slight blue‐shift (∆*λ* = 7 nm), ultimately yielding a bisignate (±) CD signal with a positive band centered at 353 nm and a negative band at 320 nm. These results indicate that **AggII** exhibits supramolecular helicity, likely influenced by temperature‐induced conformational changes of the OEG chains. Notably, increasing the temperature by only 2–3 K (*T* ≈ 327‐328 K) leads to complete loss of the CD signal, coinciding with the LCST and highlighting the narrow stability window of **AggII**. Further heating to 363 K maintains the null CD signal and induces pronounced turbidity, consistent with pronounced dehydration of the glycol chains and the simultaneous decrease in absorbance. Complementary VT‐fluorescence studies reveal a decrease in the characteristic emission band at 545 nm of **AggI** and the simultaneous emergence of a band centered at 433 nm with an additional shoulder at 545 nm upon heating to 325 K (**AggII**) (Figure 2c). Heating above the LCST induces the disappearance of the shoulder at 545 nm and results in a single emission band resembling that of the monomer species in organic solvents (see Figures 1c and 2c). VT‐NMR measurements further indicate the presence of residual monomeric species in solution above the LCST, as NMR resonances become observable above 328 K (Figure S12). Cooling the sample from 363 K back to RT restores the initial spectral features of **AggI**, whereas **AggII** is not detected at intermediate temperatures (Figure S13), most likely due to the rapid rehydration of the glycol chains.

**FIGURE 2:**
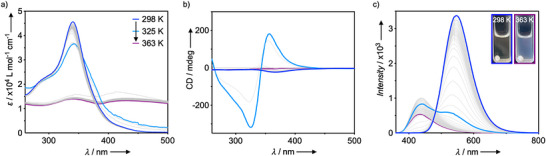
Variable‐temperature UV/Vis (a), CD (b), and emission (c) studies of **1** (*c* = 20 µM, *λ*_exc_ = 340 nm) in H_2_O. Inset in (c): solutions of **1** at 298 and 363 K illustrating the enhancement in optical density at high temperatures.

As the occurrence of LCST precluded reliable thermodynamic analysis by VT‐spectroscopy, solvent‐denaturation experiments using THF were employed instead. Analysis of the absorption changes as a function of THF volume fraction at different concentrations afforded free energies for **AggI** at 298 K (∆*G*_298 K_ = −49.40 ± 1.28 kJ mol^−1^, Figure S14) and for **AggII** at 325 K (∆*G*_325 K_ = −44.40 ± 0.86 kJ mol^−1^, Figure S15). Although determined at different temperatures and therefore not directly comparable, the similar magnitude of these values indicates that both aggregates are stabilized by comparable interaction energies within distinct thermodynamic regimes. Importantly, no interconversion of **AggI** at 298 K or **AggII** at 325 K was observed over time (Figure S16), demonstrating that each assembly corresponds to a stable supramolecular polymorphic state at the respective temperatures. Interestingly, cooling **AggII** from 325 to 298 K restores the characteristic spectroscopic features of **AggI**, evidencing that the two states are thermoreversible supramolecular polymorphs (Figure S17) [17]. Additional thermodynamic support was obtained from nano differential scanning calorimetry (nano‐DSC, Figure S18). Heating an aqueous solution of **AggI** (*c* = 1 mM) reveals a pronounced endothermic transition at approximately 327 K that coincides with the LCST, together with a minor overlapping peak that likely corresponds to the **AggI**→**AggII** polymorphic transformation.

Microscopy studies were performed to assess the impact of the LCST on the supramolecular polymorphism of **1**. Transmission electron microscopy (TEM) of **AggI** revealed discrete objects with spherical and short cylindrical morphologies and diameters of ca. 6–8 nm (Figures 3a and S20), in good agreement with aggregate heights obtained in atomic force microscopy (AFM, ca. 6 nm, Figure S19). In contrast, **AggII**, deposited at 325 K, displays micrometer‐long fiber‐like morphologies with an average width of 7 ± 1 nm (Figures 3b and S21), confirming the changes observed by spectroscopy. Imaging at 363 K showed the transformation of the elongated fibers into shorter, irregular planar assemblies once the temperature exceeded the cloud point (*T* > 325 K) [50, 51], corresponding with the formation of **AggIII** (Figures 3c and S22). Owing to the strong increase in optical density above the LCST, **AggIII** could only be identified by microscopy, as spectroscopic techniques are no longer applicable. For **AggII** and **AggIII**, the elevated temperatures required for sample preparation prevented AFM analysis. The distinct morphologies of **AggI** and **AggII** were further examined by small‐angle x‐ray scattering (SAXS) (Figure 3d–f and Table S1). Analysis of **AggI** (1 mM, 283 K) yields a satisfactory fit to a customized cylinder model with a radius of 2.35 nm (diameter = 4.7 nm) and a length of 40.3 nm (Figures 3d and S23 and Table S1). Considering the parallel slipped packing deduced from NMR and the fact that the SAXS‐derived diameter is approximately twice the length of the aromatic core of **1** (2.3 nm), **AggI** is best described as a discrete assembly composed of double antiparallel stacks with the glycol chains arranged at the periphery and pyridine units oriented toward the interior (Figures 3a,d), likely establishing hydrogen‐bonds with surrounding water molecules [52, 53]. For **AggII** (1 mM, 323 K), SAXS analysis using the same cylinder model yields a comparable radius (2.2 nm) but a markedly increased cylinder length of 429.3 nm (Figure 3e and Table S1), consistent with the formation of long 1D fibers featuring a double‐helical arrangement of **AggII** and in agreement with TEM observations (Figure 3b). SAXS measurements could not be reliably performed above the LCST (≈ 330 K at 1 mM) due to the formation of **AggIII** colloidal dispersion that prevented proper data acquisition. However, at slightly lower temperatures (323 K), a noticeable shortening of the fibers was observed, likely associated with the pre‐formation of **AggIII** (Table S1). Cooling the sample from 330 to 303 K leads to a pronounced reduction in the calculated cylinder length to 18.1 nm (Figure 3f), consistent with the direct recovery of **AggI** (Figure S17) from **AggIII** without the observation of **AggII** during the cooling process. The observed supramolecular polymorphism can be rationalized considering the temperature‐dependent reorganization and gradual dehydration of the hydrophilic side chains. At low temperatures, **AggI** is formed by direct dissolution of the bulk sample of **1** in water, where the glycol chains backfold and wrap around the hydrophobic OPE core to shield it from unfavorable interactions with the aqueous environment [37], thus preventing extended growth. Increasing the temperature slightly below the LCST enhances the conformational freedom of the glycol chains and promotes partial release of hydrogen‐bonded water molecules, resulting in a partially back‐folded conformation that allows favorable stacking of the OPE cores into extended 1D assemblies. At temperatures above the LCST, complete dehydration of the TEG chains disrupts this organization, promoting lateral association and shortening of the fibers, ultimately leading to less ordered 2D nanostructures (Scheme 1).

**FIGURE 3:**
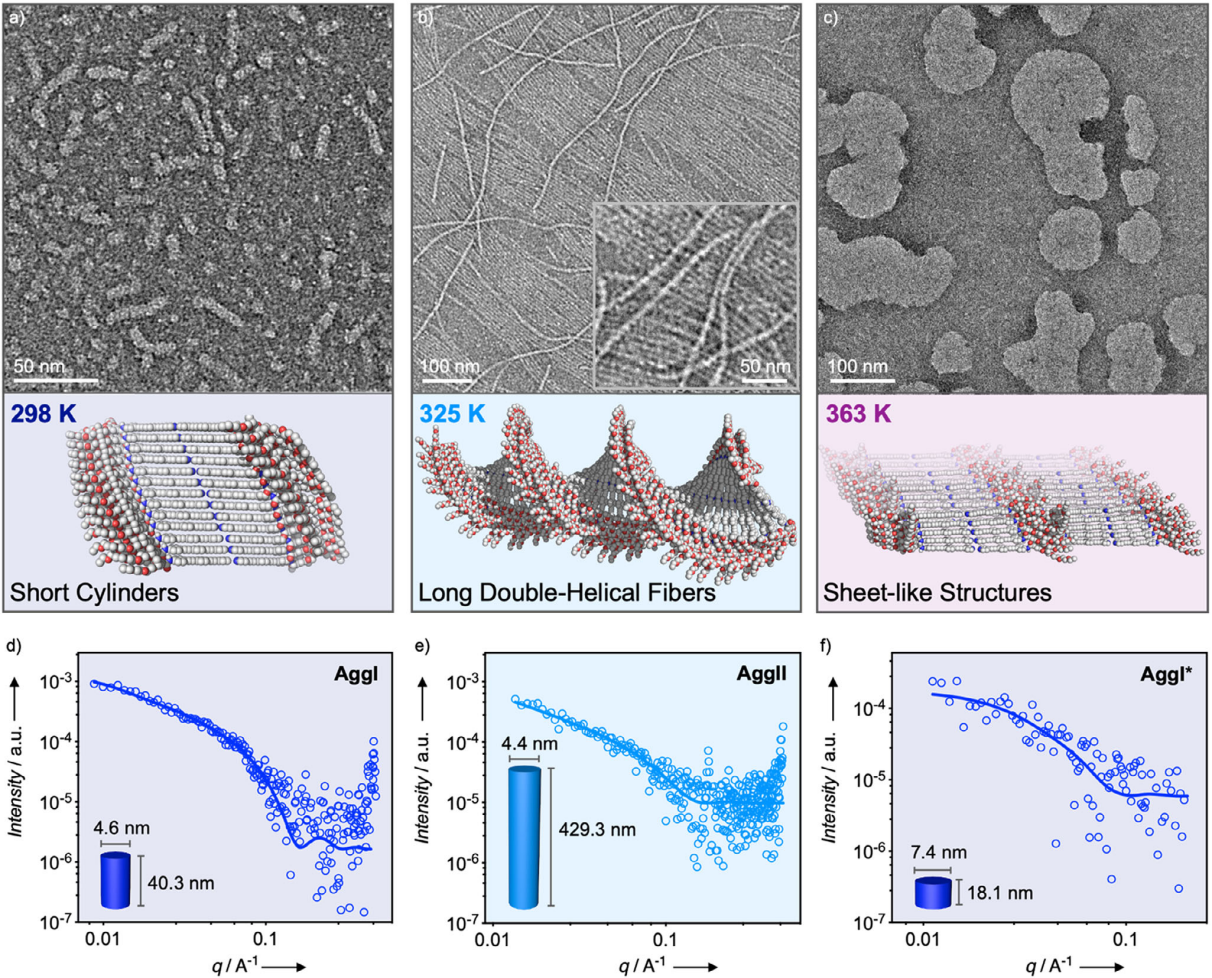
TEM images of **AggI** at 298 K (a), **AggII** at 325 K (b), and **AggIII** at 363 K (c) (*c* = 20 µM) deposited on carbon‐coated copper grids and stained with a 1% aqueous uranyl acetate solution. Experimental SAXS profiles (*c* = 1 mM, circles) recorded during heating for **AggI** at 283 K (d) and **AggII** at 323 K (e), as well as after cooling from 330 to 303 K to regenerate **AggI** (f), together with the corresponding fits (solid lines) obtained using customized models.

As the polymorphic transitions **AggI**→**AggII**→**AggIII** are governed by the LCST, fine‐tuning this parameter offers a direct means to control the supramolecular polymorphic landscape and expand the functional scope of these materials. In covalent polymers, the LCST can be modulated by adding a co‐solvent [54, 55], prompting us to investigate whether this strategy could stabilize the chiral polymorph **AggII**, which is confined to a narrow temperature window. Indeed, the addition of the water‐miscible co‐solvent acetonitrile (ACN) to an aqueous solution of **1** induces a pronounced LCST decrease of 25 K, reaching 300 K for a H_2_O:ACN 85:15 (v/v) mixture. At low ACN fractions (*x*_ACN_ < 0.15), the supramolecular system remains largely unaffected (Figure S24) owing to the formation of hydration cages around the co‐solvent molecules. At higher ACN contents, collapse of these hydration structures [56, 57] enables direct ACN‐glycol interactions, disrupting the local water network and promoting the **AggI**→**AggII** transition near RT, thereby allowing isolation of **AggII** (Figure 4a,b). In parallel, we examined the influence of ionic strength on the hydrogen‐bonding interactions between the glycol chains and water [55, 58, 59, 60]. Interestingly, gradual addition of NaCl or KCl (0–2.3 M) shifts the **AggI**→**AggII** transition to lower temperatures, decreasing from 325 to 302 K for Na^+^ and to 306 K for K^+^ (Figures 4c and S25–S27). A comparable ion‐specific trend is observed for the anions, with Cl^−^ exerting a stronger effect than Br^−^, while I^−^ shows negligible influence, likely due to its larger ionic radius and weaker hydration capability (Figures 4c, S25 and S26). The differences in transition temperatures observed for the various ions can be rationalized by the Hofmeister series [59], which describes the relative ability of ions to stabilize or destabilize hydrated structures. Notably, shifting the formation temperature of **AggII** by the use of ACN as a co‐solvent enabled its morphological characterization by AFM at RT, thereby overcoming the previous limitations associated with sample preparation at elevated temperatures. AFM imaging discloses the formation of (*P*)‐oriented double‐helical fibers with an average height of ca. 7–8 nm and several microns in length and with a pronounced tendency to bundle (Figures 4d,e and S28). On this basis, we constructed a 3D model integrating all structural insights obtained from spectroscopy and microscopy, revealing that the molecules adopt a slight rotational offset to relieve steric hindrance imposed by the lateral chains (Figure 4f,g). This proposed packing is additionally validated for a 24‐mer using theoretical calculations at the (GFN2‐xTB) level (Figure S29a,b) [61]. Moreover, TD‐DFT (rCAM‐B3LYP/3‐21G) [62, 63, 64] calculations performed on a tetramer extracted from the xTB‐calculated 24‐mer stack allowed us to obtain theoretical UV/Vis and ECD spectra in good agreement with the experimental data (Figure S29c,d). Overall, the use of external stimuli highlights the adaptability of the system and underscores the central role of the LCST in governing polymorphic transitions, offering a powerful strategy for tailoring supramolecular architectures.

**FIGURE 4:**
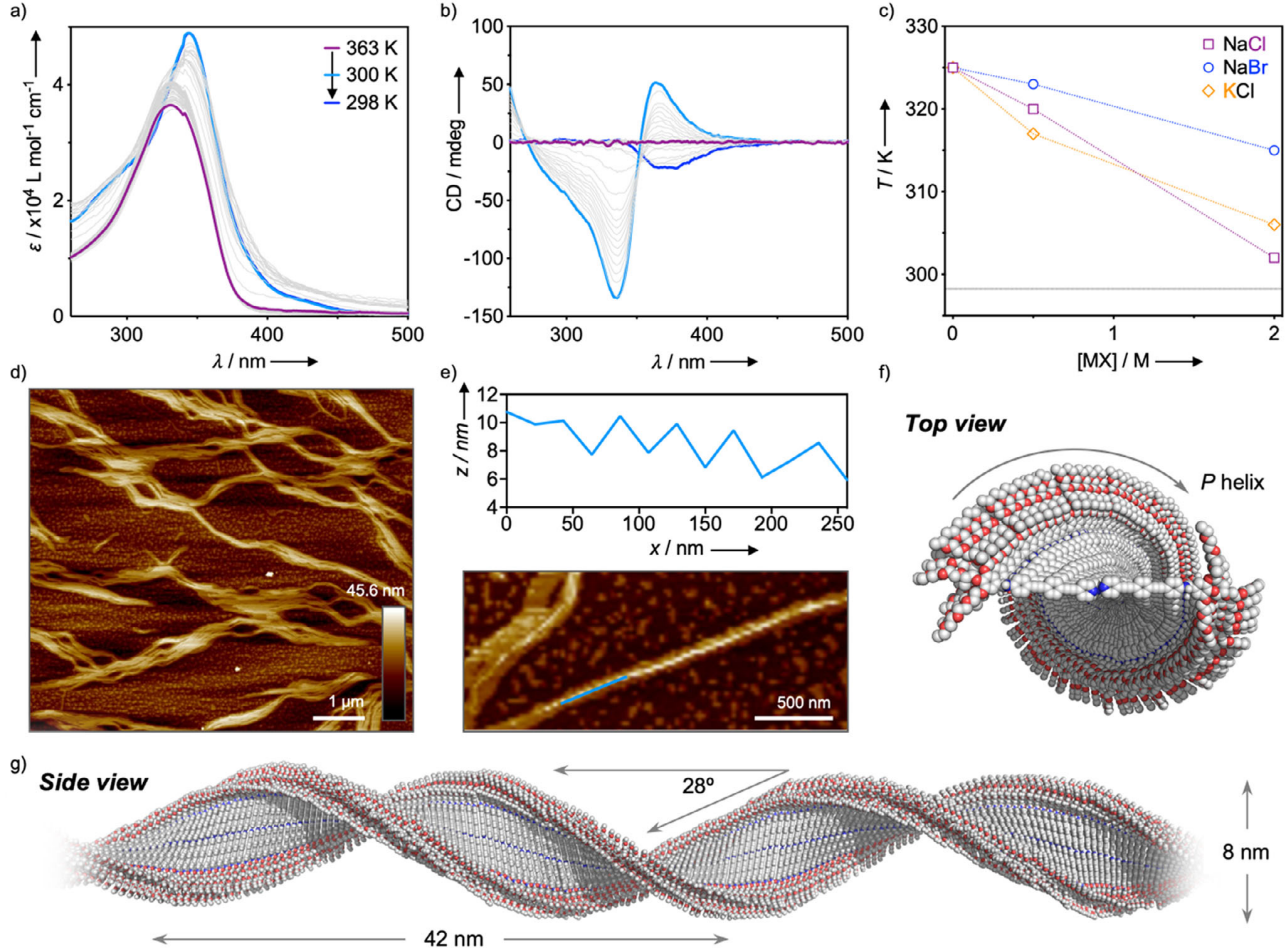
VT‐UV/Vis (a) and CD (b) spectra of **1** in H_2_O:ACN 85:15 (v/v) (*c* = 20 µM). c) Variation of the *T*_cloud_ of **1** (*c* = 20 µM) as a function of salt concentration in aqueous solutions of NaCl (purple), NaBr (blue), and KCl (orange). (d, e) AFM images of **AggII** on mica obtained after spin‐coating (1500 rpm) a H_2_O:ACN 85:15 (v/v) solution of **1** cooled from 363 to 300 K (*c* = 20 µM). The inset in (e) shows the height profile along the blue line. Top (f) and side (g) views of the 3D model reconstructed from AFM data, highlighting the pitch, packing angle, and polymer width of **AggII**.

In conclusion, we demonstrate a distinctive example of chiral supramolecular polymorphism in aqueous media, where precise temperature control governs transitions and stabilizes three distinct polymorphs with different morphologies and photophysical properties. At RT, **AggI** is formed through hydrophobic interactions, adopting a discrete morphology in which backfolded glycol chains effectively shield the hydrophobic core from the aqueous environment, preventing extended growth. Further heating slightly below the LCST induces a structural reorganization into helical fibers (**AggII**) driven by partial dehydration and backfolding of the glycol chains. Above the LCST, a third polymorph (**AggIII**) with lower order and planar morphology emerges, demonstrating the temperature‐dependent tunability of these supramolecular assemblies. Remarkably, adjusting chemical stimuli such as co‐solvent composition and ionic strength allows isolation of the otherwise narrow temperature‐window helical polymorph **AggII** at RT, enabling full control over the polymorphic landscape. Our findings broaden the understanding of thermally controlled supramolecular polymorphs and offer new insights into the design of chiral and adaptive supramolecular materials in aqueous media.

## Conflicts of Interest

The authors declare no conflict of interest.

## Supporting information




**Supporting File 1**: The authors have cited additional references within the Supporting Information [62–69].

## Data Availability

The data that support the findings of this study are available from the corresponding author upon reasonable request.
