# Supplementary material for: Thermally Controlled Chiral Supramolecular Polymorphism in Water
Source: Angew Chem Int Ed Engl. 2026 May 2;65(25):e1190401. doi: 10.1002/anie.1190401 (PMC13266910; doi:10.1002/anie.1190401)
Supplement: Supplementary file 1 — Supporting File 1: The authors have cited additional references within the Supporting Information [62–69]. [file ANIE-65-e1190401-s001.docx]

Supporting Information

**Thermally Controlled Chiral Supramolecular Polymorphism in Water**

Zulema Fernández,* ^[a]^ Yongsheng Li,^[b]^ Daniel Martínez,^[c]^ Julia Terlau,^[a]^ Myongsoo Lee,^[b]^ Bartolomé Soberats,^[c]^ Gustavo Fernández^[a]^

[a] Universität Münster, Organisch-Chemisches Institut Corrensstraße 36, 48149, Münster, Germany
E-mail: fernandz@uni-muenster.de

[b] Department of Chemistry, State Key Lab of Molecular Engineering of Polymers and Shanghai Key Lab of Molecular Catalysis and Innovative Materials, Fudan University, 200438, Shanghai, China

[c] Department of Chemistry, Universitat de les Illes Balears, Cra. Valldemossa km 7.5, 07122, Palma de Mallorca, Spain

*: corresponding author

**Abstract:** Biological assemblies such as proteins adapt their helical morphology and function in response to external stimuli, yet controlled polymorphic transitions in synthetic chiral supramolecular analogues remain poorly understood. Herein, we demonstrate a strategy to achieve controlled chiral supramolecular polymorphism in water by coupling molecular design with external stimuli. An unsymmetrical oligo(phenyleneethynylene) derivative **1** bearing a pyridine unit, a hydrogen-bonding amide group and chiral hydrophilic side chains self-assembles into three distinct chiral supramolecular polymorphs in water that are stable at different temperature regimes. At room temperature (RT), **1** self-assembles into short cylinders (**AggI**), which undergo a polymorphic transition to transient double helical fibers upon heating around the LCST (**AggII**, ≈ 325 K) and ultimately to irregular planar aggregates (**AggIII**) above the LCST. Remarkably, the polymorphic transitions are linked to the temperature-dependent conformation and degree of dehydration of the glycol chains. Although **AggII** exists only within a narrow temperature window in pristine water, it can be stabilized and isolated at RT through chemical stimuli such as co-solvents or metal salts that modulate the LCST. Our results establish LCST-coupled chirality as a powerful strategy to regulate thermoresponsive supramolecular polymorphism and offer potential strategies for the design of adaptive materials.

**Table of Contents**

[1. Experimental Section 2](#_Toc221787183)

[1.1. Materials and Methods 2](#_Toc221787184)

[1.2. Synthesis and Characterization 4](#_Toc221787185)

[2. Supplementary Figures 10](#_Toc221787186)

[3. References 24](#_Toc221787187)

# 1. Experimental Section

## 1.1. Materials and Methods

**General Procedures**

All solvents were dried according to standard procedures. Reagents were used as purchased. All air sensitive reactions were carried out in Schlenk tubes under argon atmosphere, using Teflon-coated magnetic stirring bars.

For spectroscopic measurements quartz cuvettes with optical pathlengths of 0.01, 0.1 and 1 cm were employed and spectroscopic grade solvents were used. The sample concentration and temperature are indicated for each experiment.

**Chemicals and Reagents**

All chemicals were purchased from Merck (Darmstadt, Germany), TCI Europe N.V. (Tokyo, JP), BLD Pharm (Shanghai, China) or Alfa Aesar (Ward Hill, MA, USA), with minimum analytical grade quality and used without further purification.

**Column Chromatography**

Preparative column chromatography was performed in self-packed glass columns of different sizes with silica gel (VWR silica gel 60, particle size 40-63 µm). Pentane, ethyl acetate, dichloromethane and methanol were distilled before use.

**NMR Spectroscopy**

^1^H and ^13^C NMR experiments were recorded at 298 K on a Bruker NEO 400 (^1^H: 400 MHz; ^13^C: 100.6 MHz). 2D ^1^H^1^H-COSY and ^1^H-ROESY were acquired using an Agilent DD2 600 (^1^H: 600 MHz). All experiments were performed in deuterated solvents and referenced to the remaining resonance signals (CDCl_3_: 7.26 ppm (^1^H); DCM: 5.32 ppm (^1^H); D_2_O: 4.79 ppm (^1^H)). The chemical shifts (*δ*) observed are given with respect to the chemical shift of the trimethylsilane (0 ppm). The spin multiplicities of the proton signals are abbreviated as *s* (singlet), *d* (doublet)*, t* (triplet), *dd* (doublet of doublets) and *m* (multiplet*)*.

**Mass Spectrometry (MS)**

ESI mass spectra were recorded on a Bruker MicroToF instrument with loop injection. The signals are described by their mass/charge ratio (*m*/*z*) in Da.

**UV/Vis Spectroscopy**

All UV/Vis spectra were measured on a JASCO V-770 spectrophotometer, equipped with a Peltier cell and a Julabo F250 water circulation unit. The data were recorded with a spectral bandwidth of 2.0 nm and a scanning speed of 1000 nm min^-1^. The sample concentration is indicated for each experiment.

**CD Spectroscopy**

CD spectra were recorded on a JASCO J-1500 spectrophotometer, equipped with a Peltier cell and a Julabo F250 water circulation unit. The data were recorded with a spectral bandwidth of 1.0 nm and a scanning speed of 500 nm min^-1^. The sample concentration is indicated for each experiment.

**Fluorescence Spectroscopy**

Fluorescence experiments were performed on a JASCO FP-8500 spectrofluorimeter, equipped with a Peltier cell and a Julabo F250 water circulation unit. The sample concentration is indicated for each experiment.

**Atomic Force Microscopy (AFM)**

AFM images were acquired using a **Multimode® 8 SPM system** (Bruker AXS). **AC200TS cantilevers** (Oxford Instruments) were employed (average spring constant: **9 N m⁻¹;** resonance frequency: **150 kHz;** length: **200 µm;** width: **40 µm;** nominal tip radius: **7 nm**). Samples were **spin-coated onto freshly cleaved mica** and measured **the following day** to ensure complete solvent evaporation. Measurement conditions for each sample are specified below.

**Transmission Electron Microscopy (TEM)**

A 5 μL aliquot of the sample solution was deposited onto a carbon-coated copper grid (Carbon Type B, 15-25 nm, on 200-mesh Cu with Formvar; Ted Pella, Inc.). After 1 min, excess solution was wicked away with filter paper. The grid was then negatively stained with 10 μL of an aqueous uranyl acetate solution (1.0 wt%) and air-dried. TEM images were collected on a JEOL 2100 Plus microscope operated at 200 kV using an SC1000 CCD camera (Gatan, Inc., Warrendale, PA). Images were recorded and processed using DigitalMicrograph (v3.60.4441.0).

**Small Angle X-ray Scattering (SAXS)**

SAXS measurements were performed on a XENOCS XEUSS 3.0. The instrument is equipped with a GeniX 3D Cu micro focus X-ray source (λ = 1.54 Å; flux = 2 × 10^8^ ph/s) and a DECTRIS Eiger X 1M detector. Experiments were performed using the temperature controlled BIOCUBE module (2 mm capillary) with a sample-to-detector distance of 600 mm. Measurement times were 3600 s in all cases.

For sample preparation, compound **1** was directly dissolved in of MilliQ water (previously filtered through 0.22 mm PVDF filter) (*c* ~ 1 mM) and measured using BioCUBE module.

Background subtraction (against the solvent) was carried out using the XSACT software (Xenocs). The subtracted curves were fitted to customized models using the software SASView.^1^ The curves were fitted to distinct models and the best fittings (χ²) results are shown in Table S1.

**Theoretical Calculations**

Theoretical calculations were performed by means of the xTB 6.6.0 program package.^2^ Full geometry optimizations were carried out at the cost-effective semiempirical GFN2-xTB level of theory, method which is based on a Hamiltonian similar to the well-known DFTB3, with a minimal valence basis set centered on atoms (STO-mG), and includes the density-dependent D4 dispersion correction.^3^

TD-DFT calculations were carried out in combination with the rCAM-B3LYP functional and 3-21G basis set (SCRF = H_2_O). The ECD and UV/Vis calculations were performed including 80 excitation energies on previously xTB-optimized tetramers. The full width at half height (FWHM) was fixed to 15.0 nm and the ECD were plotted with Gaussian curves.

Models were done in Avogadro 1.2.0^4^ and the optimized structures represented in Pymol 2.5.2.

**1.2. Synthesis and Characterization**

Compounds **I**^5^ **III**,^6^ **IV**^6^ and **X**^7^ were obtained following previously reported procedures. The spectroscopic characterization agrees with that previously reported in the literature.

**Figure S1.** Synthesis of (*S*)-chiral compound **1**.

Synthesis of (*S*)-12-methyl-2,5,8,11-tetraoxatridecan-13-yl-4-methylbenzenesulfonate (**V**)

**IV** (1.40 g, 6.29 mmol, 1.00 equiv) was introduced in a previously purged Schlenk tube and dissolved in DCM (15.0 mL, degassed/dry). Next, the solution was cooled down to 0 ºC and *p*-toluenesulfonyl chloride (TsCl, 1.80 g, 9.44 mmol, 1.50 equiv), 4-dimethylaminopyridine (DMAP, 0.15 g, 1.25 mmol, 0.20 equiv) and Et_3_N (2.62 mL, 19.0 mmol, 3.00 equiv, degassed/dry) were added. The reaction mixture was allowed to warm up to room temperature and further stirred for two days at this temperature. The crude was washed with citric acid conc. (2 x 50 mL), NaHCO_3_ conc. (2 x 50 mL) and dried over MgSO_4_. The solvent was evaporated and the crude was purified by flash chromatography (silica; EtOAc/Pentane 70/30) to afford **V** as a colorless oil (2.00 g, 83%).

**^1^H NMR** (400 MHz, CDCl_3_, 298 K) δ (ppm) = 7.78 (d, *J* = 8.2 Hz, 2H), 7.33 (d, *J* = 8.2 Hz, 2H), 3.93 (m, 2H), 3.69 (m, 1H), 3.64–3.50 (m, 13H), 3.36 (s, 3H), 2.43 (s, 3H), 1.11 (d, 3H).

**^13^C NMR** (150 MHz, CD_2_Cl_2_) δ (ppm) = 144.8, 132.8, 129.8, 129.3, 127.9, 126.5, 73.4, 72.6, 71.8, 70.6, 70.5, 70.4, 70.3, 68.8, 21.6, 21.4, 16.6, 16.6.

**MS** (ESI) m/z calculated for [M+Na] =399.1448, found = 399.1445.


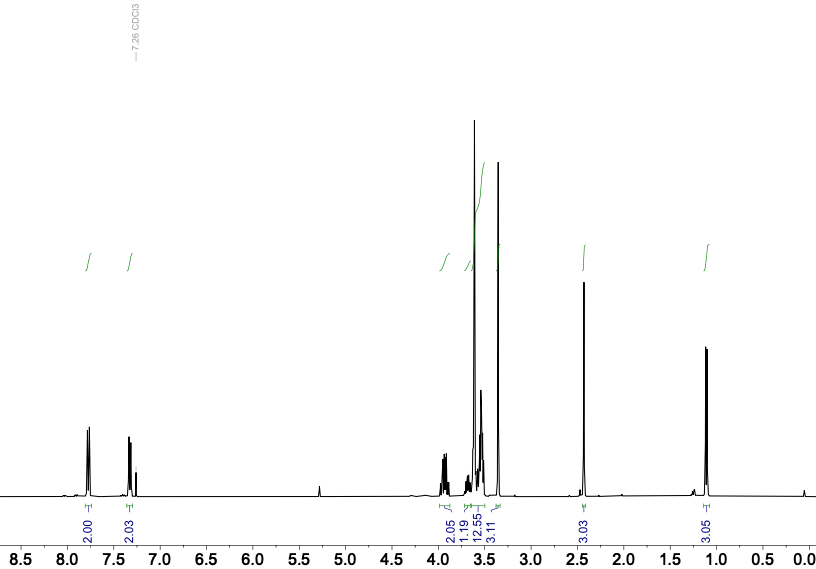


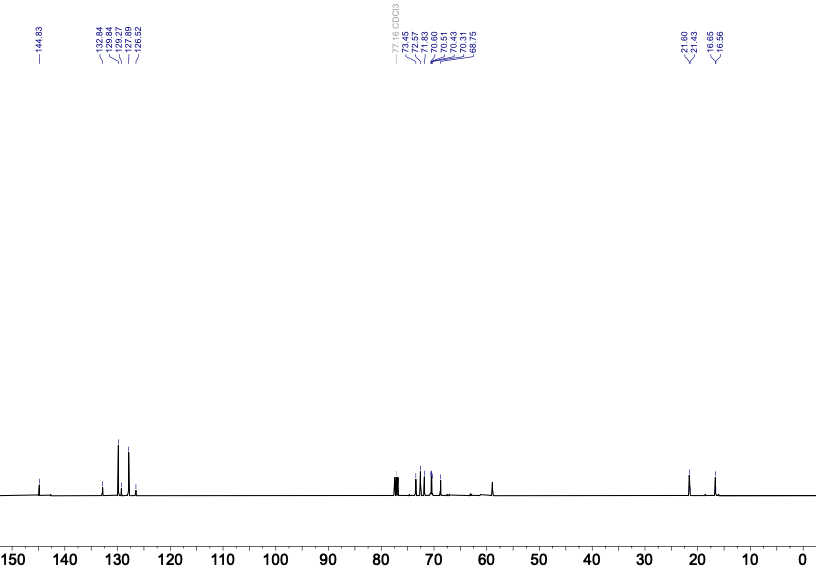


**Figure S2.** ^1^H NMR (400 MHz, CDCl_3_, 298 K) (top) and ^13^C NMR (150 MHz, CD_2_Cl_2_, 298 K) (bottom) spectra for compound **V**.

Synthesis of methyl 3,4,5-tris(((*S*)-12-methyl-2,5,8,11-tetraoxatridecan-13-yl)oxy)benzoate (**VII**)

**V** (1.40 g, 3.71 mmol, 3.30 equiv) and methyl 3,4,5-trihydroxybenzoate (**VI**, 0.21 g, 1.13 mmol, 1.00 equiv) were introduced in a previously purged Schlenk tube and dissolved in DMF (15.0 mL, degassed/dry). Next, K_2_CO_3_ (0.78 g, 5.63 mmol, 5.00 equiv) was added and the mixture was stirred overnight at 90 ºC. The crude was subsequently washed with distilled water (2 x 50 mL) and dried over MgSO_4_. The solvent was evaporated and the crude was purified by flash chromatography (silica; DCM/EtOH 97/3) to afford **VII** as a yellowish oil (0.70 g, 77%).

**^1^H NMR** (400 MHz, CDCl_3_, 298 K) δ (ppm) = 7.25 (s, 2H), 4.10–4.01 (m, 3H), 3.93–3.77 (m, 9H), 3.72–3.60 (m, 30H), 3.54–3.50 (m, 6H), 3.35 (s, 9H), 1.31–1.25 (m, 9H).

**^13^C NMR** (150 MHz, CD_2_Cl_2_) δ (ppm) = 166.6, 152.3, 142.1, 129.8, 128.0, 124.9, 108.3, 76.3, 75.0, 74.3, 71.9, 70.8, 70.6, 70.6, 70.5, 68.8, 68.6, 59.0, 52.2, 17.5.

**MS** (ESI) m/z calculated for [M+Na] = 819.4349, found = 819.4335.


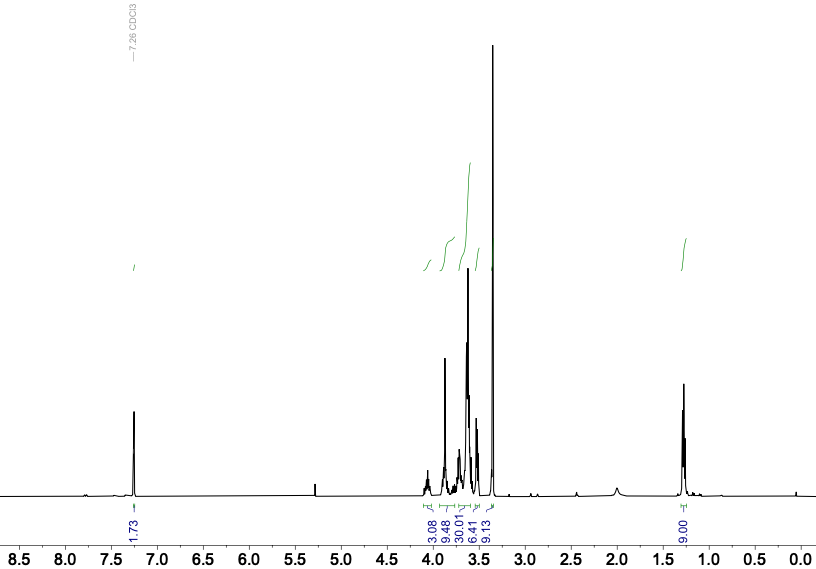


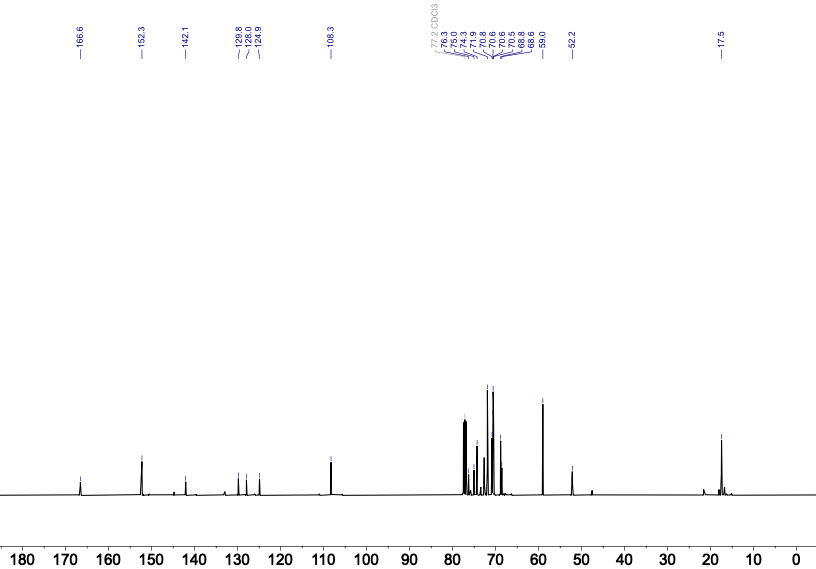


**Figure S3.** ^1^H NMR (400 MHz, CDCl_3_, 298 K) (top) and ^13^C NMR (150 MHz, CD_2_Cl_2_, 298 K) (bottom) spectra for compound **VII**.

Synthesis of 3,4,5-tris(((*S*)-12-methyl-2,5,8,11-tetraoxatridecan-13-yl)oxy)benzoic acid (**VIII**)

**VII** (0.70 g, 0.87 mmol, 1.00 equiv) was introduced in a round bottom flask and dissolved in EtOH (3.00 mL). Next, KOH (0.15 g, 2.63 mmol, 3.00 equiv) was dissolved in distilled water (3.00 mL) and added to the previous solution. The mixture was stirred overnight under reflux. The crude was evaporated, redissolved in distilled water (20.0 mL) and acidified to pH = 1. The product was extracted with DCM (2 x 30.0 mL) and the solvent was evaporated to afford **VIII** as a colorless oil (0.67 g, 97%) that was used without further purification.

**^1^H NMR** (400 MHz, CDCl_3_, 298 K) δ (ppm) = 7.32 (s, 2H), 4.12–4.03 (m, 3H), 3.93–3.78 (m, 6H), 3.71–3.59 (m, 30H), 3.55–3.51 (m, 6H), 3.36 (d, 9H), 1.27 (dd, *J* = 6.3, 2.4 Hz, 9H).

**^13^C NMR** (150 MHz, CD_2_Cl_2_) δ (ppm) = 166.6, 152.3, 142.1, 129.8, 128.0, 124.9, 108.3, 76.3, 75.0, 74.3, 71.9, 70.8, 70.6, 70.5, 70.5, 68.8, 68.6, 59.0, 17.5.

**MS** (ESI) m/z calculated for [M+Na] = 805.4192, found = 805.4193.


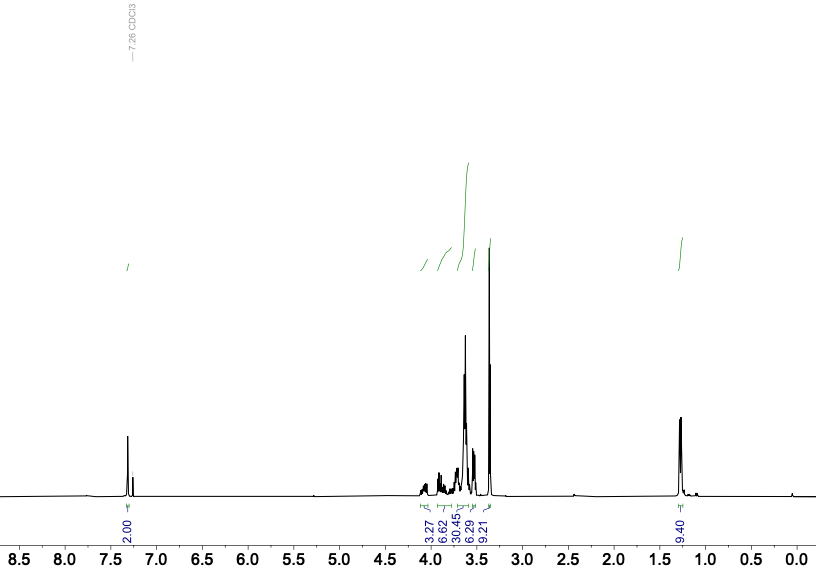


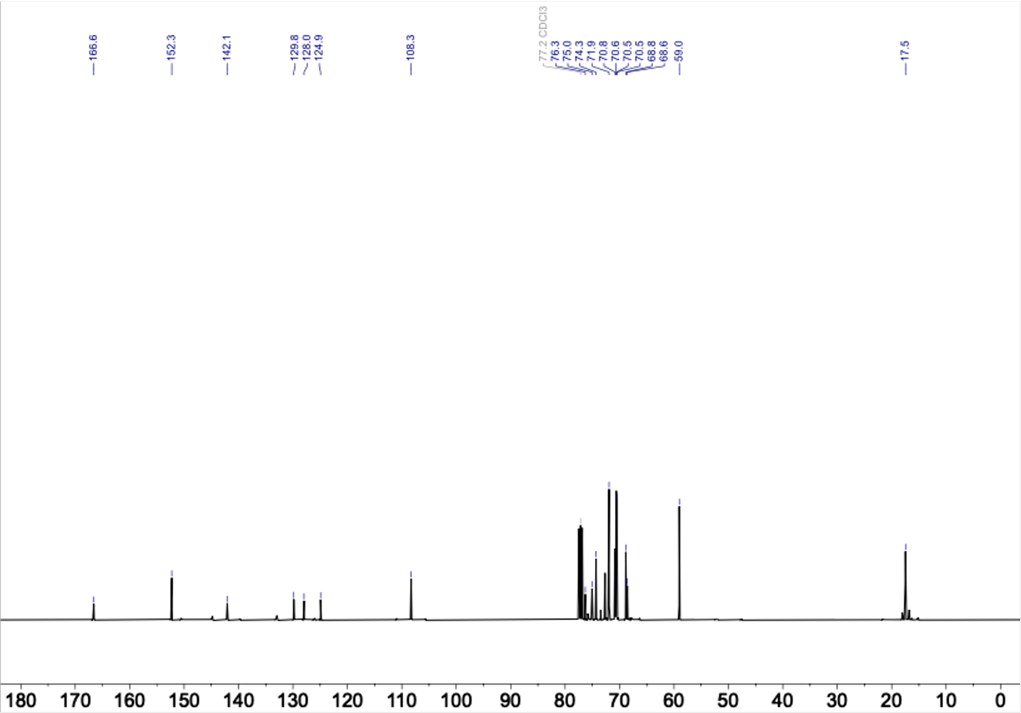


**Figure S4.** ^1^H NMR (400 MHz, CDCl_3_, 298 K) (top) and ^13^C NMR (150 MHz, CD_2_Cl_2_, 298 K) (bottom) spectra for compound **VIII**.

Synthesis of *N*-(4-iodophenyl)-3,4,5-tris(((*S*)-12-methyl-2,5,8,11-tetraoxatridecan-13-yl)oxy)benzamide (**IX**)

4-iodoaniline (0.19 g, 0.85 mmol, 1.00 equiv) was introduced in a previously purged Schlenk tube and dissolved in DCM (10.0 mL, degassed/dry). **VIII** (0.67 g, 0.85 mmol, 1.00 equiv), DMAP (0.07 g, 0.60 mmol, 0.70 equiv) and 1-Ethyl-3-(3-dimethylaminopropyl)carbodiimide (EDC; 0.40 g, 0.26 mmol, 3.00 equiv) were added and the reaction was stirred overnight at room temperature. The solvent was evaporated and the crude was purified by flash chromatography (silica; DCM/MeOH 98/2) to afford **IX** as a colorless oil (0.70 g, 83%).

**^1^H NMR** (400 MHz, CDCl_3_, 298 K) δ (ppm) = 8.76 (s, 1H), 7.61 (d, *J* = 8.7 Hz, 2H), 7.48 (d, *J* = 8.8 Hz, 2H), 7.23 (s, 2H), 4.08–4.02 (m, 3H), 3.89–3.57 (m, 36H), 3.53–3.46 (m, 6H), 3.31 (d, *J* = 25.8 Hz, 9H), 1.25 (m, 9H).

**^13^C NMR** (150 MHz, CD_2_Cl_2_) δ (ppm) = 165.7, 152.7, 138.6, 137.9, 130.0, 122.5, 107.8, 87.5, 76.5, 75.2, 74.6, 73.4, 72.1, 72.0, 71.0, 70.7, 70.7, 70.6, 70.6, 69.0, 68.8, 68.7, 59.2, 59.1, 59.1, 17.3.

**MS** (ESI) m/z calculated for [M+Na] =1006.3632, found = 1006.3638.


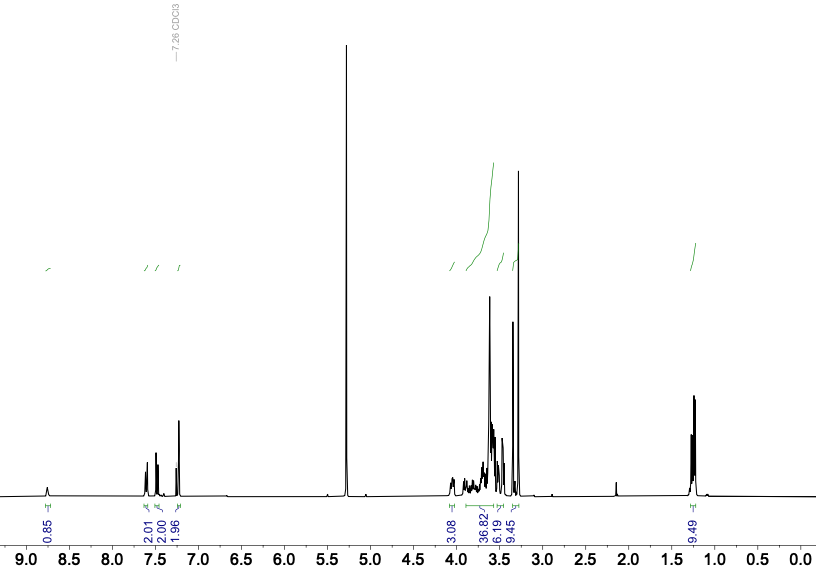


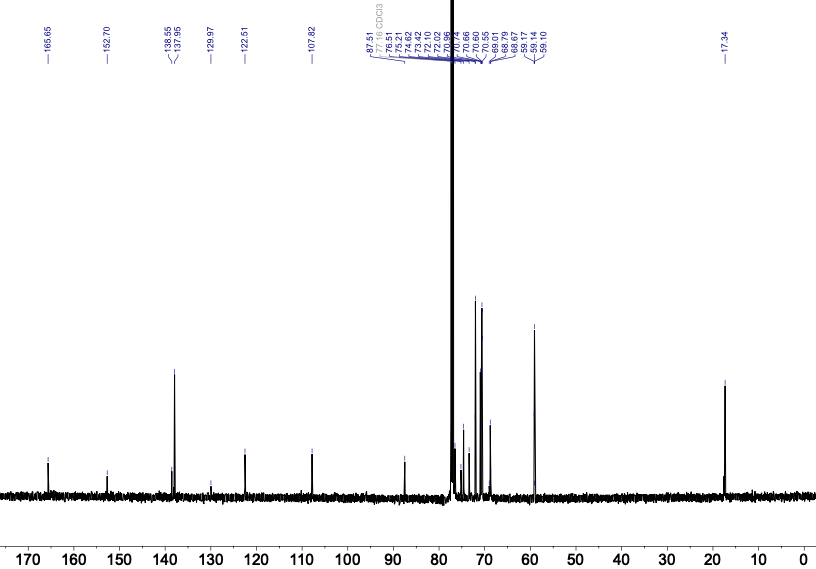


**Figure S5.** ^1^H NMR (400 MHz, CDCl_3_, 298 K) (top) and ^13^C NMR (150 MHz, CD_2_Cl_2_, 298 K) (bottom) spectra for compound **IX**.

Synthesis of 3,4,5-tris(((*S*)-12-methyl-2,5,8,11-tetraoxatridecan-13-yl)oxy)-*N*-(4-((4-(pyridine-4-ylethynyl)phenyl)ethynyl)phenyl)benzamide (**1**)

**IX** (0.30 g, 0.60 mmol, 0.70 equiv), Pd(PPh_3_)_4_ (0.01 g, 0.01 mmol, 0.02 equiv) and CuI (0.01 g, 0.01 mmol, 0.02 equiv) were introduced in a previously purged Schlenk tube and dissolved in THF (15.0 mL, degassed/dry). Next, Et_3_N (10.0 mL, degassed/dry) and **X** (0.06 g, 0.30 mmol, 1.00 equiv) were added and the reaction was stirred overnight at room temperature. The solvent was evaporated and the crude was purified by flash chromatography (silica; DCM/MeOH 98/2) to afford **1** as a yellowish oil (0.20 g, 63%).

**^1^H NMR** (400 MHz, CD_2_Cl_2_, 298 K) δ (ppm) = 8.78 (s, 1H), 8.59 (dd, *J* = 6.0, 2.7 Hz, 2H), 7.78–7.74 (m, 2H), 7.57–7.52 (m, 6H), 7.40 (dd, *J* = 6.1, 2.8 Hz, 2H), 7.27 (s, 2H), 4.12–4.06 (m, 3H), 4.02–3.91 (m, 3H), 3.89–3.77 (m, 3H), 3.76–3.66 (m, 6H), 3.61–3.54 (m, 24H), 3.51–3.45 (m, 6H), 3.31 (d, *J* = 28.1 Hz, 9H), 1.28 (dd, *J* = 6.3, 2.1 Hz, 9H).

**^13^C NMR** (150 MHz, CD_2_Cl_2_) δ (ppm) = 165.8, 153.0, 150.3, 141.9, 139.7, 138.1, 132.7, 132.3, 131.9, 131.4, 130.3, 125.8, 124.7, 122.8, 122.1, 120.6, 118.4, 110.5, 107.5, 93.6, 92.2, 88.7, 88.7, 76.7, 75.4, 74.8, 73.5, 72.3, 72.3, 71.2, 70.9, 70.9, 70.8, 70.8, 70.8, 70.7, 69.0, 69.0, 68.8, 59.0, 59.0, 17.5, 17.4.

**MS** (ESI) m/z calculated for [M+Na] = 1081.5248, found = 1081.5242.


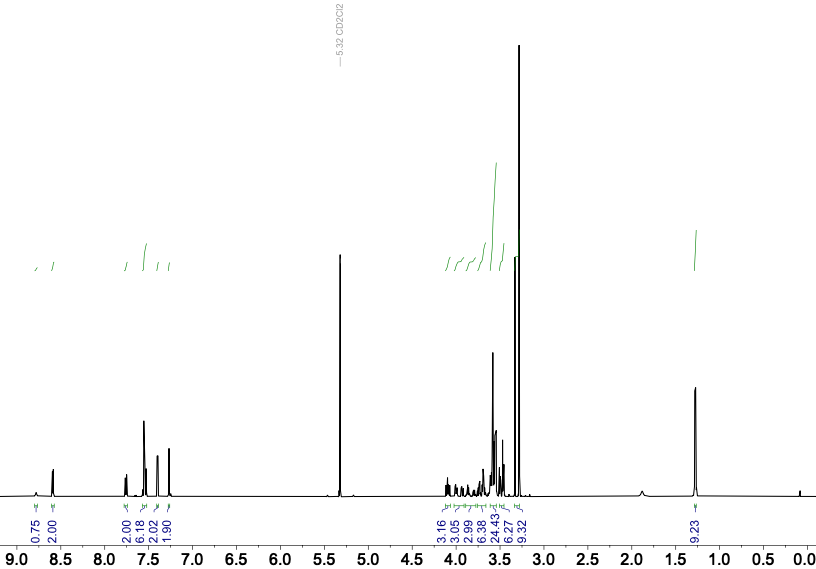


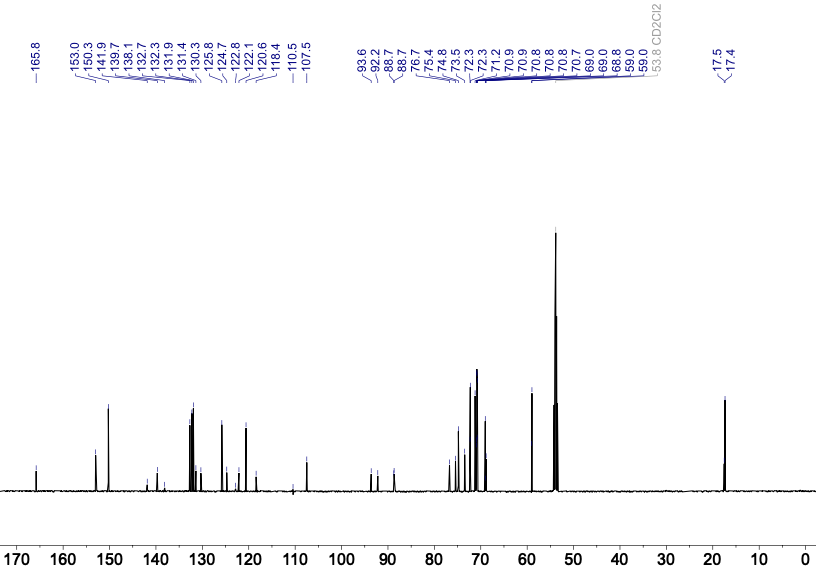


**Figure S6.** ^1^H NMR (400 MHz, CD_2_Cl_2_, 298 K) (top) and ^13^C NMR (150 MHz, CD_2_Cl_2_, 298 K) (bottom) spectra for compound **1**.

#
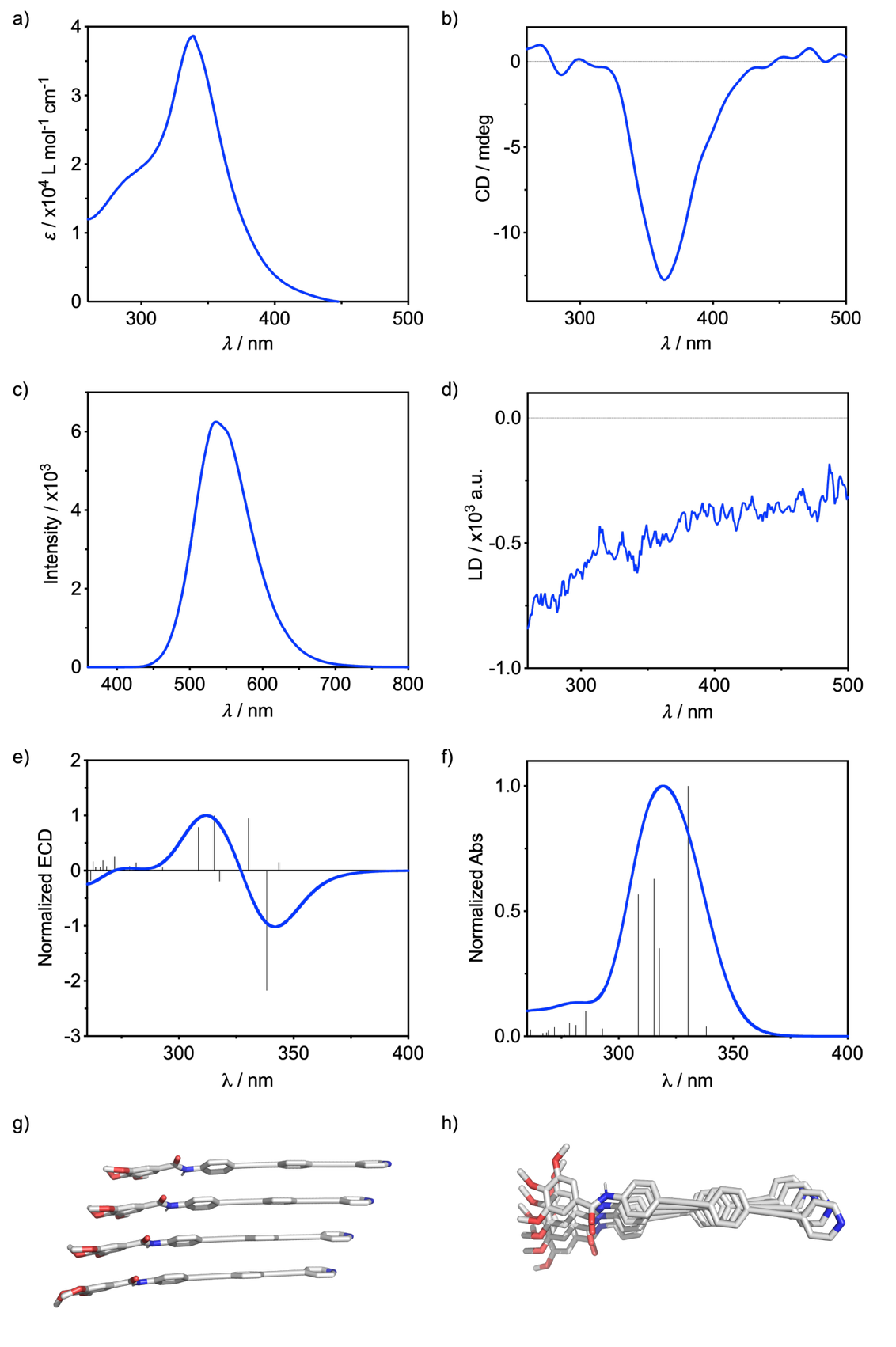
2. Supplementary Figures

**Figure S7.** UV/Vis (a) CD (b), emission (c) and LD (d) spectra of an aqueous solution of **AggI** (*c* = 20 µM, 298 K). TD-DFT (rCAM-B3LYP/3-21G) calculated CD (e) and UV/Vis (f) spectra of **AggI**, based on a tetramer previously optimized at the xTB level. Side (g) and top (h) view of an optimized tetramer stack of **AggI**. The initial geometry was constructed based on experimental data extracted from spectroscopic studies. To reduce computational costs, the tetraethylene glycol chains were replaced by methyl groups and the optimized structure was obtained using the GFN2-xTB method.


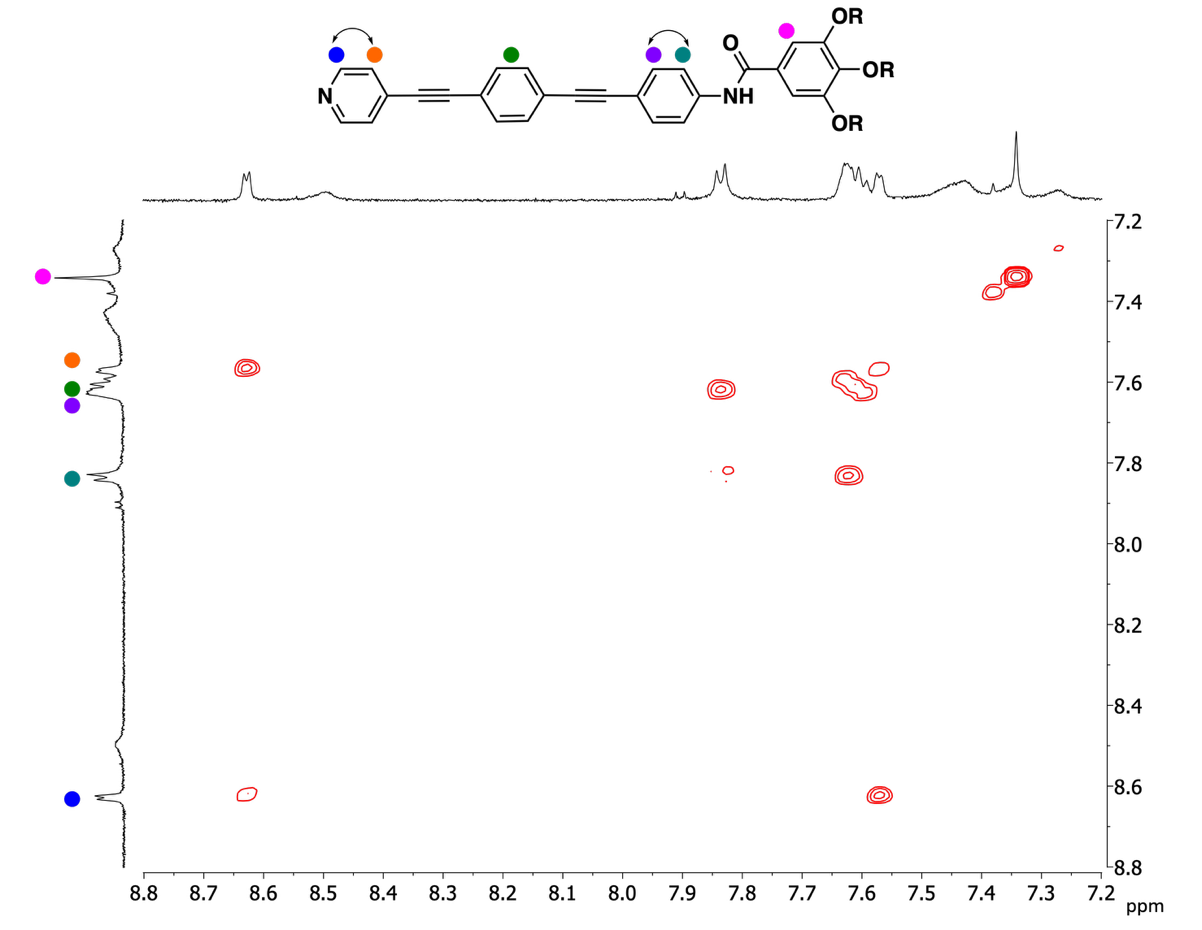
**Figure S8.** ^1^H^1^H-COSY NMR spectrum of **1** (600 MHz, *c* = 1 mM, D_2_O:CD_3_CN 70:30 (v/v), 299 K).


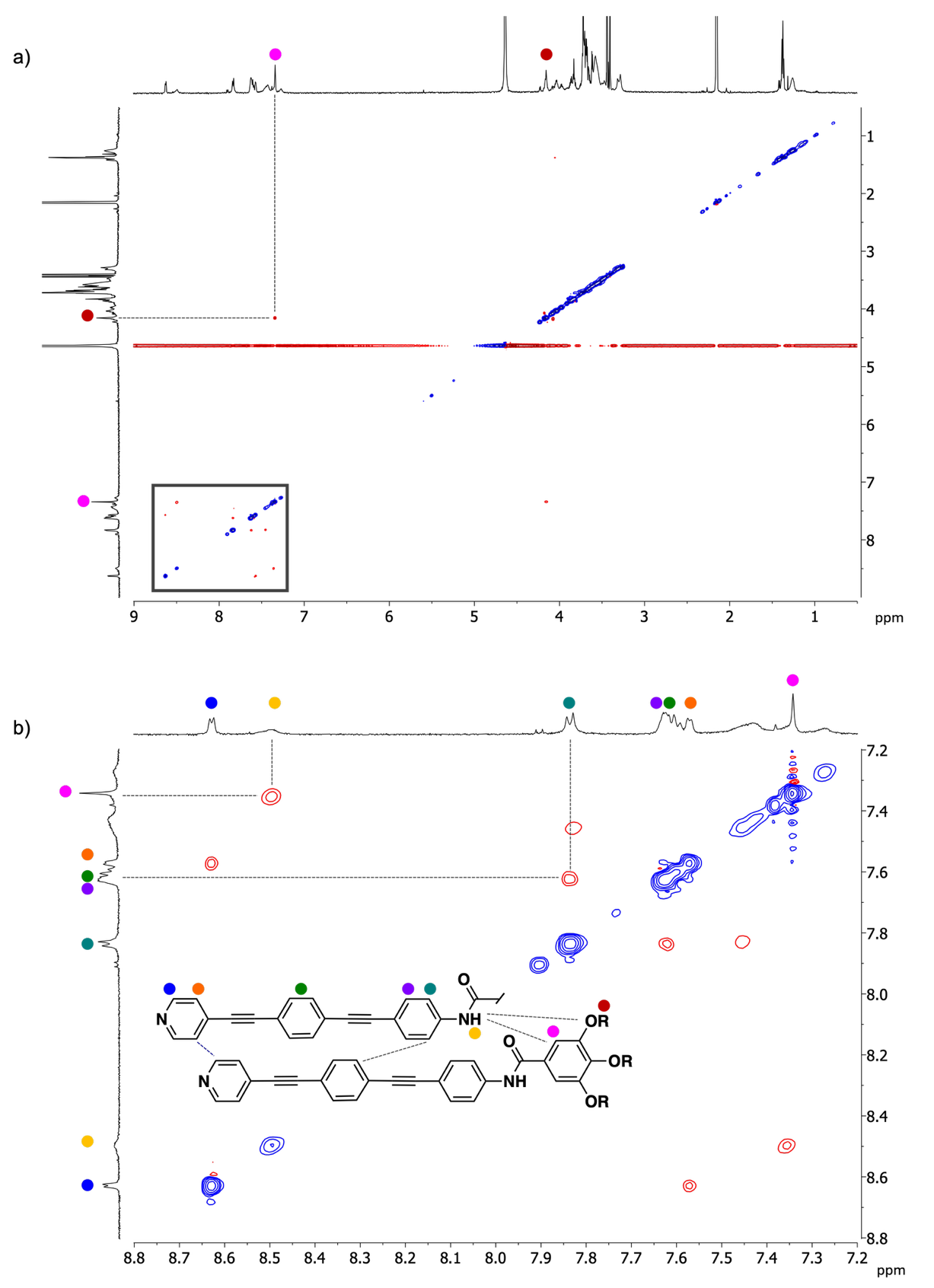


**Figure S9.** a) ^1^H-ROESY NMR spectrum of **1** (600 MHz, *c* = 1 mM, D_2_O:CD_3_CN 70:30 (v/v), 299 K). b) Zoom of the highlighted area in a) with the proposed packing arrangement and corresponding intermolecular interactions.


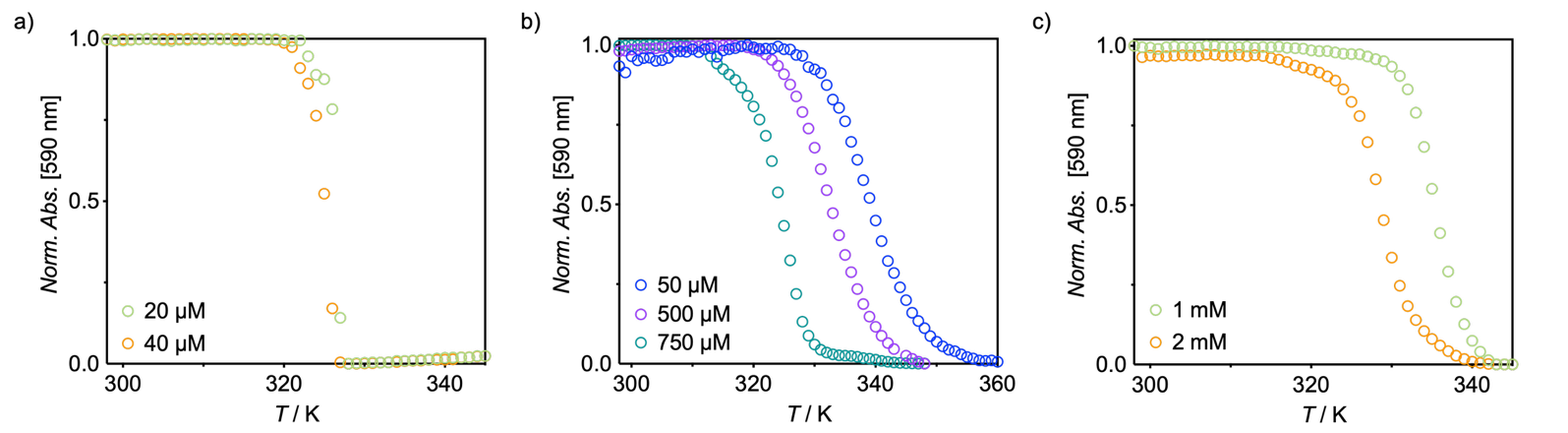


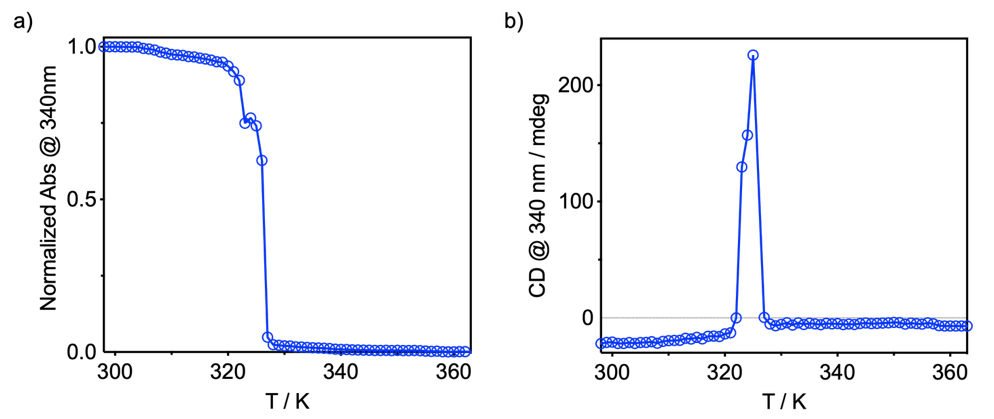
**Figure S10.** Plot of absorbance vs temperature recorded at 590 nm for different concentrations: a) 20 and 40 µM (10 mm cuvette); b) 50, 500 and 750 µM (1 mm cuvette) and c) 1 and 2 mM (0.1 mm cuvette). This representation demonstrates the dependency of the LCST on the temperature, which is shifted to lower temperatures with increasing concentrations.


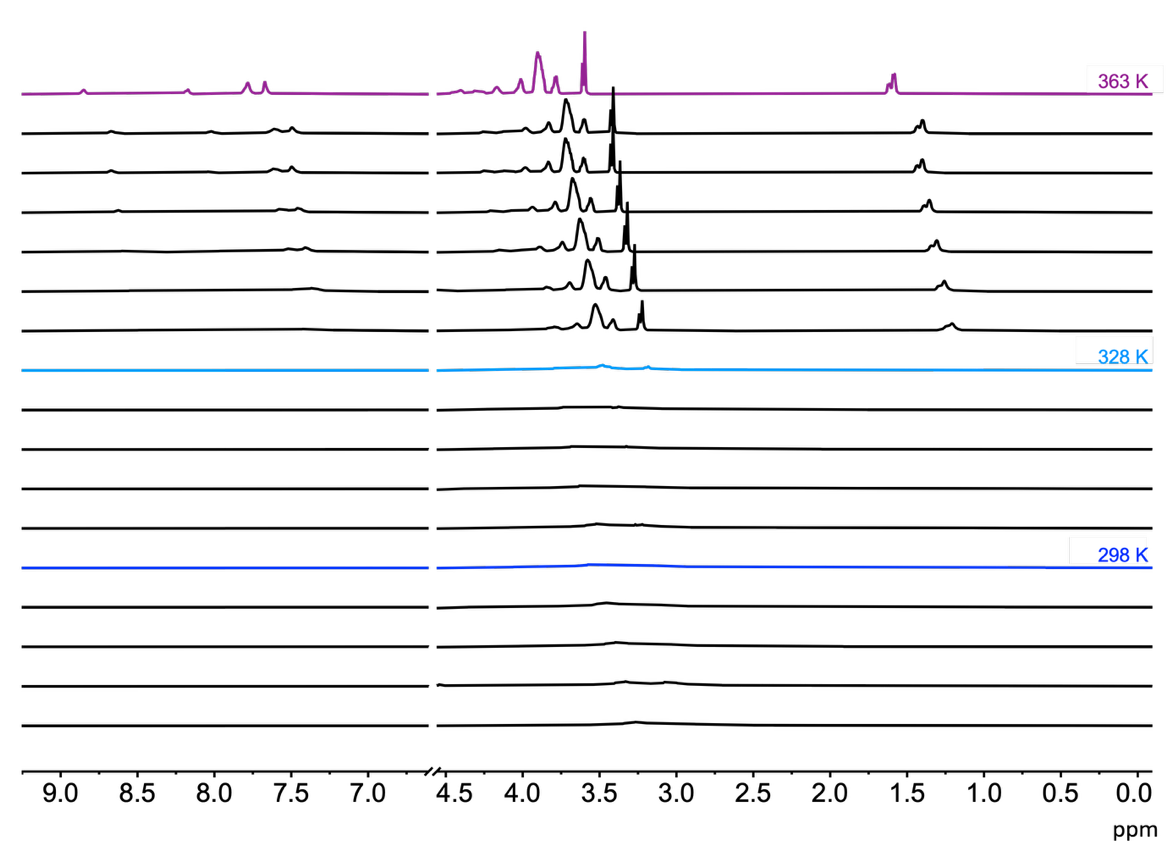
**Figure S11.** Plot of the absorbance (a) and CD (b) vs temperature monitored at 340 nm (*c* = 20 µM). In UV/Vis, a clear transition point can be observed at 325 K, matching the maximum in CD ascribed to the formation of **AggII**.

**Figure S12.** VT-NMR of **1** in D_2_O (*c* = 1 mM). Upon decreasing the temperature, a shielding of the aromatic resonances is observed due to the self-assembly driven by aromatic and hydrophobic interactions. Cooling below 328 K results in extended aggregation.


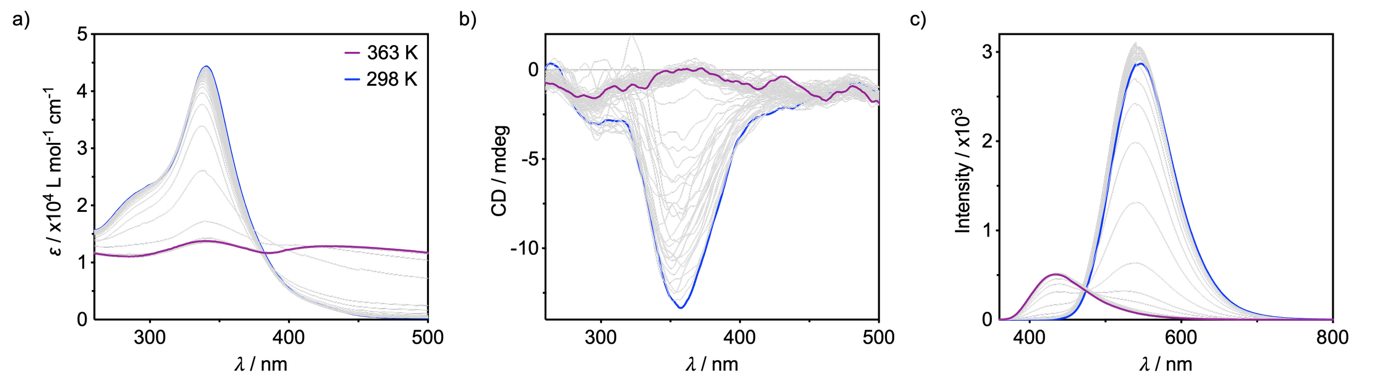


**Figure S13.** Cooling UV/Vis (a), CD (b) and emission (c) spectra of **1** from 363 K to 298 K (*c* = 20 µM, H_2_O). Upon cooling, the formation of **AggII** is not observed as an intermediate state towards **AggI**.


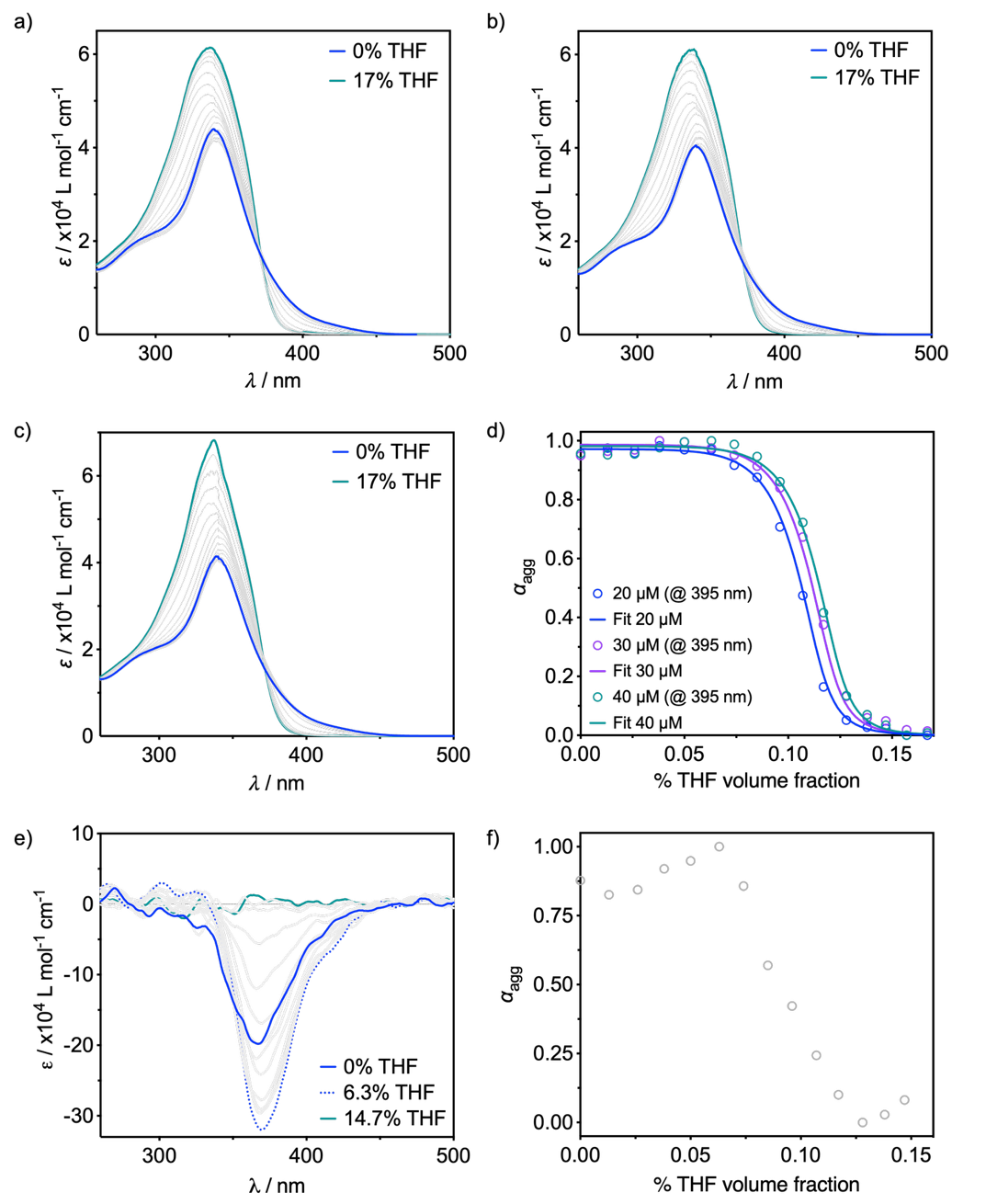


**Figure S14.** UV/Vis denaturation studies of **AggI** recorded at 298 K in H_2_O:THF mixtures at different concentrations: a) 20 µM, b) 30µM and c) 40 µM. d) Corresponding denaturation curves obtained at 395 nm and fitted to the nucleation-elongation model.^8^ CD denaturation studies of **AggI** recorded at 298 K in H_2_O:THF mixtures at 20 µM (e) and corresponding secondary plot showing the variation of the CD signal with increasing amounts of THF (f). The absence of an initial plateau prevents fitting the curve to the nucleation-elongation model to extract the thermodynamic parameters.


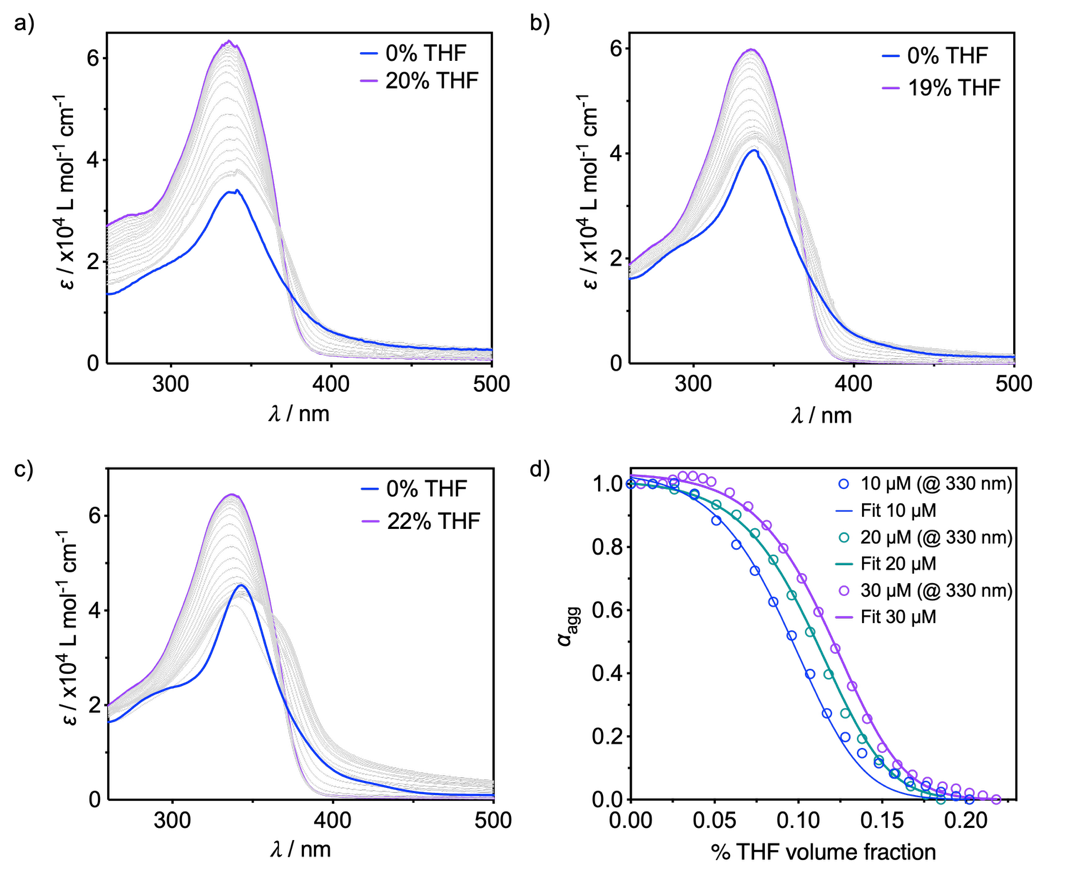


**Figure S15.** UV/Vis denaturation studies of **AggII** recorded at 325 K in H_2_O:THF mixtures at different concentrations: a) 10 µM, b) 20 µM and c) 30 µM. d) Corresponding denaturation curves obtained at 330 nm and fitted to the nucleation-elongation model.^8^


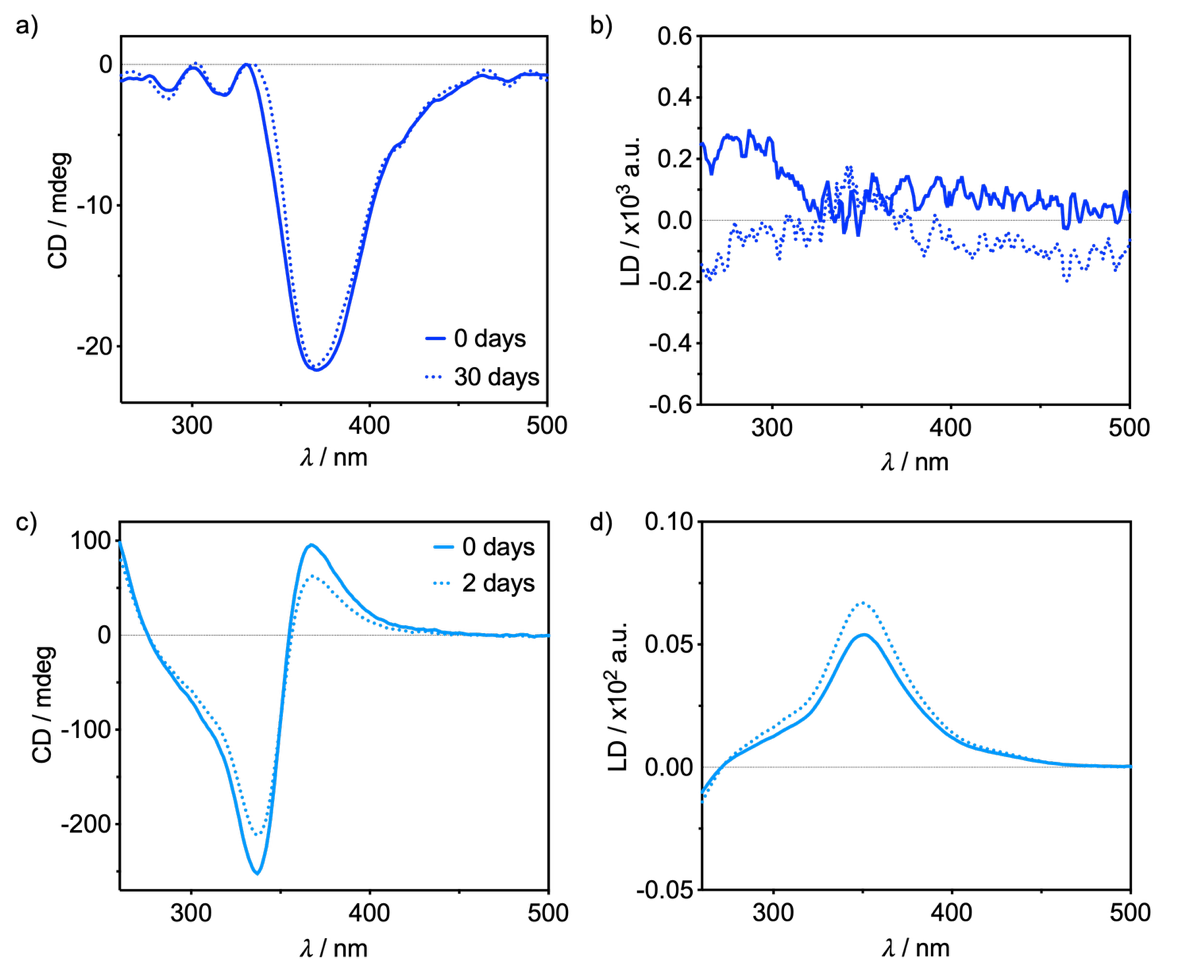


**Figure S16.** CD (a) and LD (b) studies for **AggI** of a freshly prepared sample and the same sample measured after 30 days at 298 K (*c* = 20 µM). CD (c) and LD (d) studies for **AggII** of a freshly prepared sample and the same sample measured after 2 days at 325 K (*c* = 20 µM). The lack of spectroscopic changes when recording the sample over time indicates that the aggregates are thermoreversible supramolecular polymorphs.

**
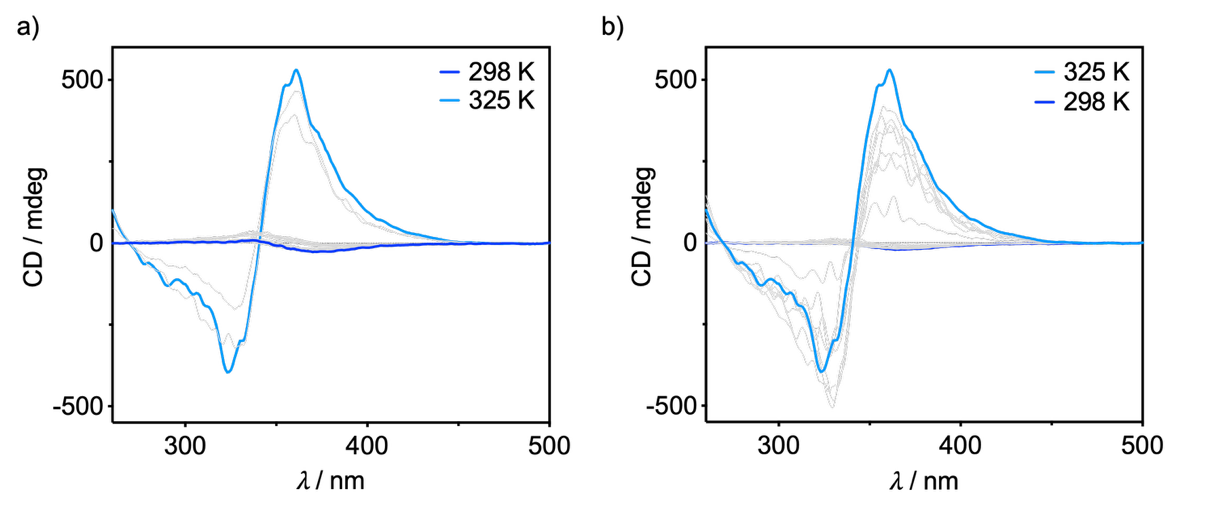
**

**
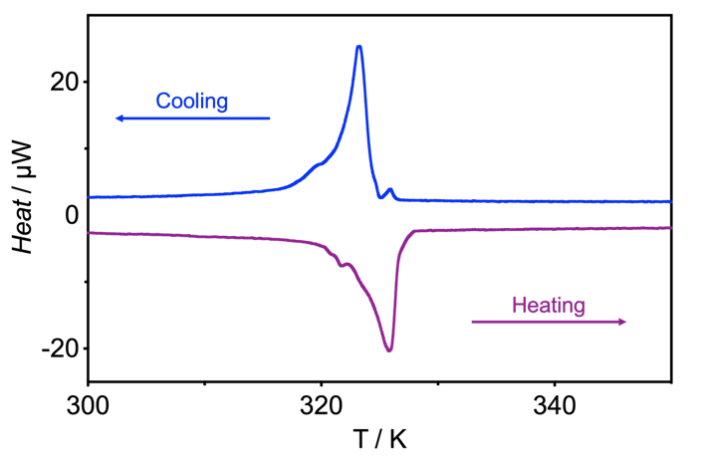
Figure S17.** Heating (a) and cooling (b) CD spectra. Upon heating to 325 K, slightly below the LCST, **AggI** transforms into **AggII**. Cooling the sample back to RT results in recovery of the initial spectral features.


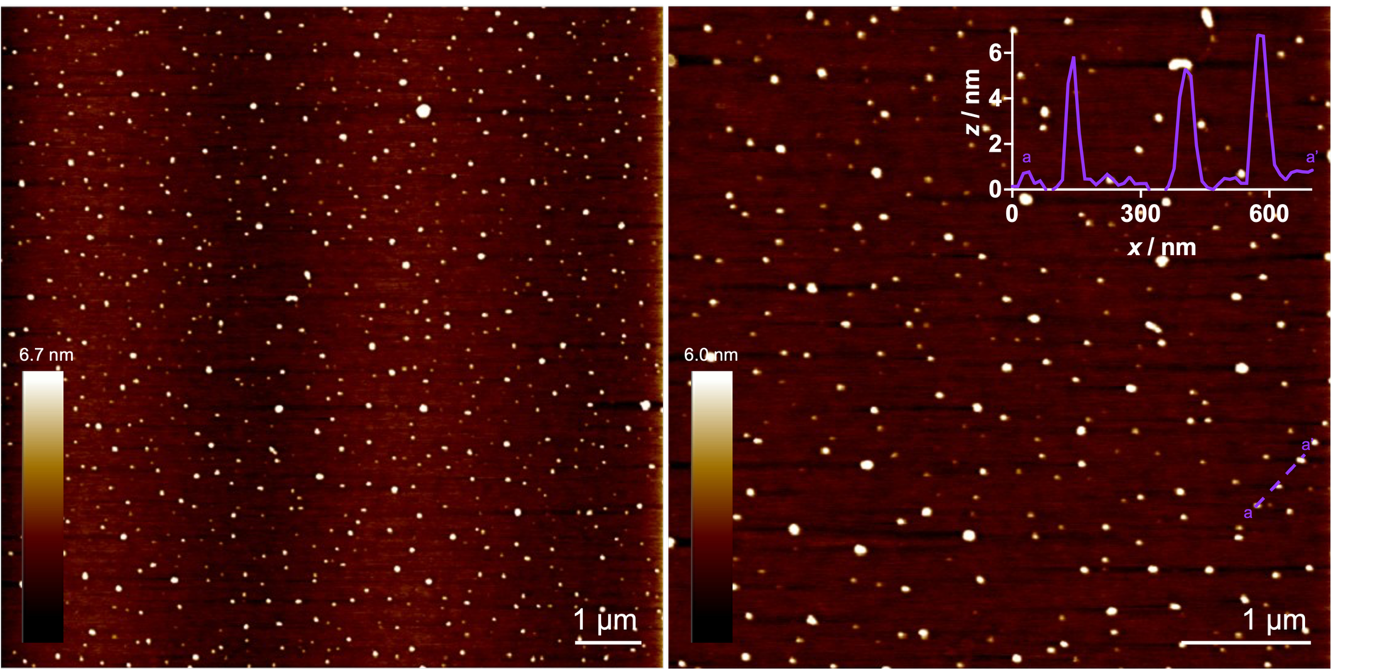
**Figure S18.** Nano-DSC thermograms showing the second heating (purple) and cooling (blue) cycle (H_2_O, *c* = 1 mM; 1 K·min^-1^). The sharp endothermic peak observed at 325 K upon heating corresponds to the LCST transition and is in close agreement with the value determined by spectroscopy studies (*T*_LCST_ = 328 K). The slight discrepancy is likely due to differences in concentration and sample volume used in the experiment that shifts the LCST to lower temperatures (Figure S9). The shoulder observed prior to this transition is attributed to the **AggI** to **AggII** transformation, mediated by the dehydration of the glycol chains. Remarkably, cooling the sample exhibits full reversibility. Because this process is coupled to the LCST, a quantitative thermodynamic analysis is not feasible; nevertheless, integration of the transition yields an overall enthalpy change of (ΔH) of 63.52 kJ mol⁻¹.

**Figure S19.** AFM images on mica obtained from a spin-coated solution of **AggI** (*c* = 20 µM, 100 rpm, 298 K, H_2_O). The inset displays the height profile along the purple line.

**
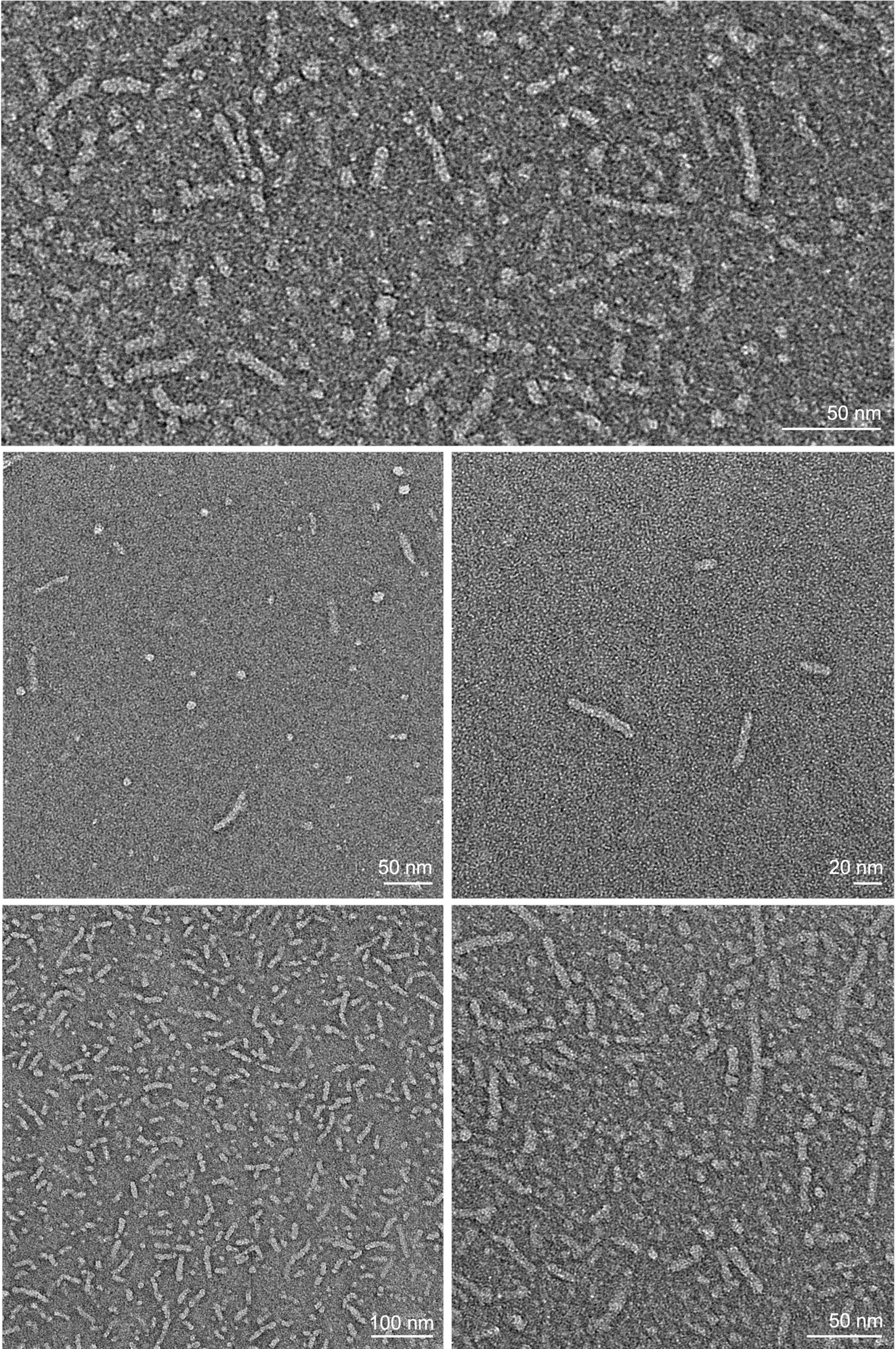
**

**Figure S20.** TEM images of **AggI** (*c* = 20 µM, 298 K, H_2_O) deposited on a carbon-coated copper grids and stained with a 1% aqueous solution of uranyl acetate.

**
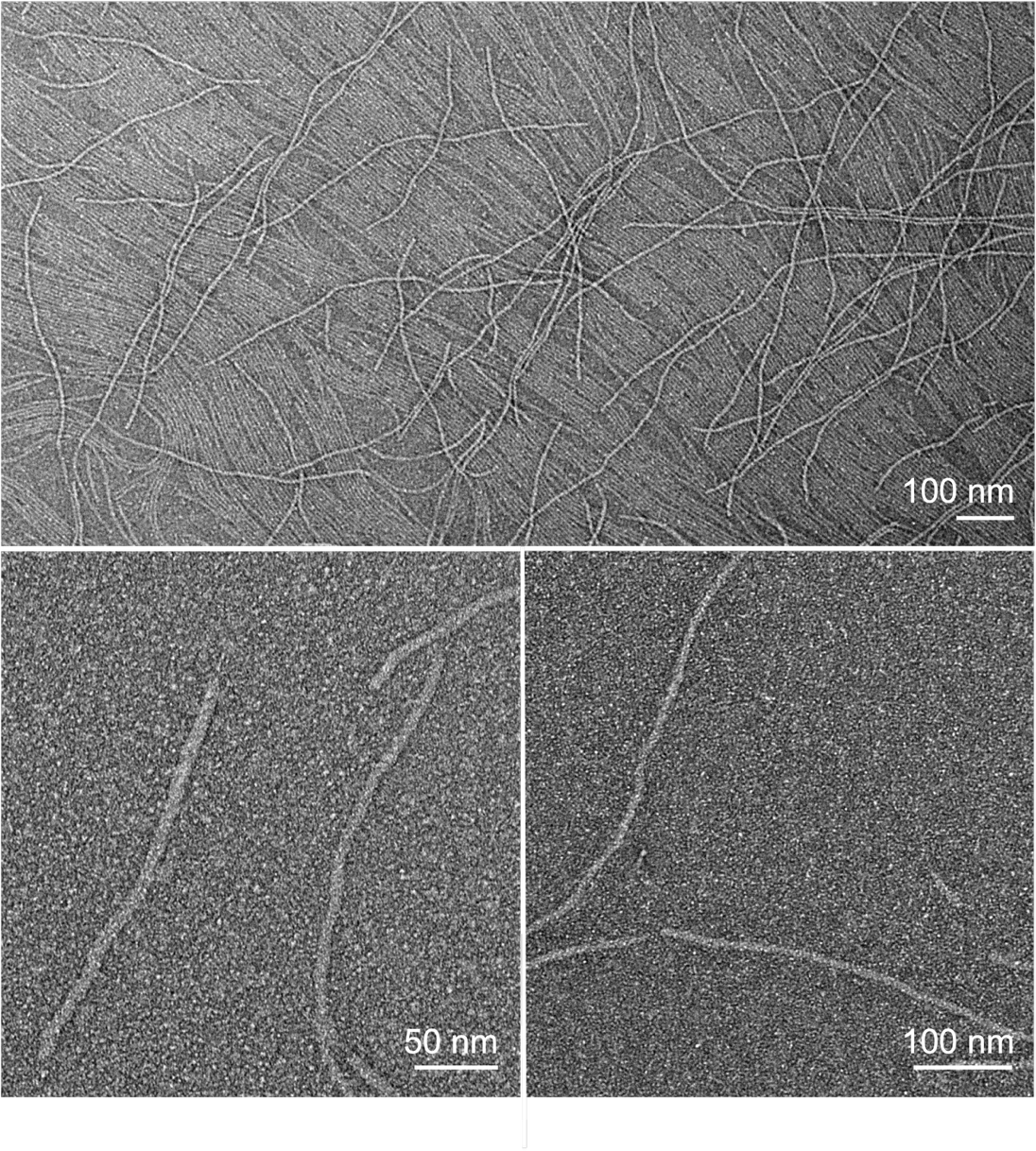
Figure S21.** TEM images of **AggII** (*c* = 20 µM, 325 K, H_2_O) deposited on a carbon-coated copper grids and stained with a 1% aqueous solution of uranyl acetate.


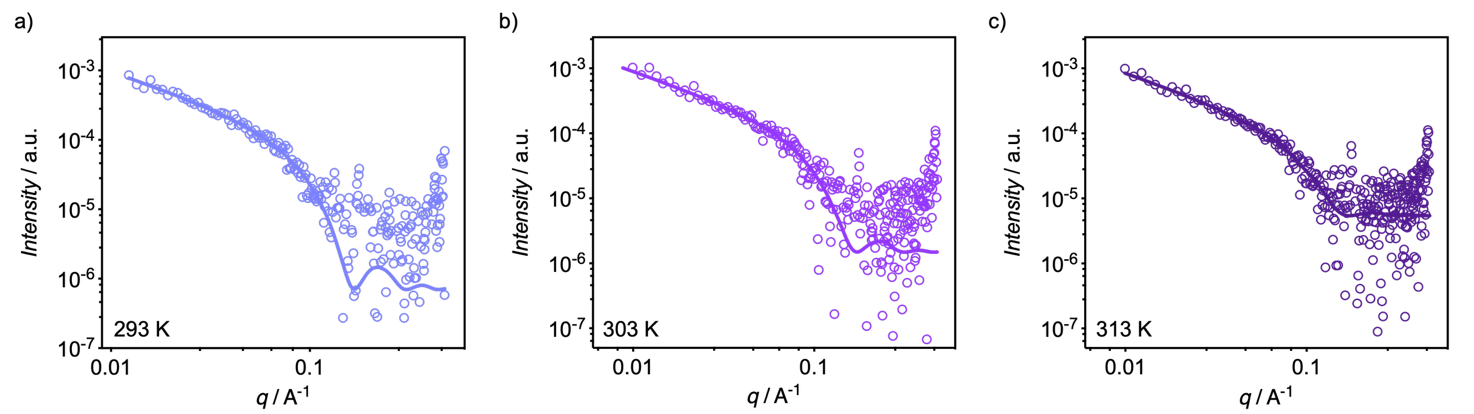
**
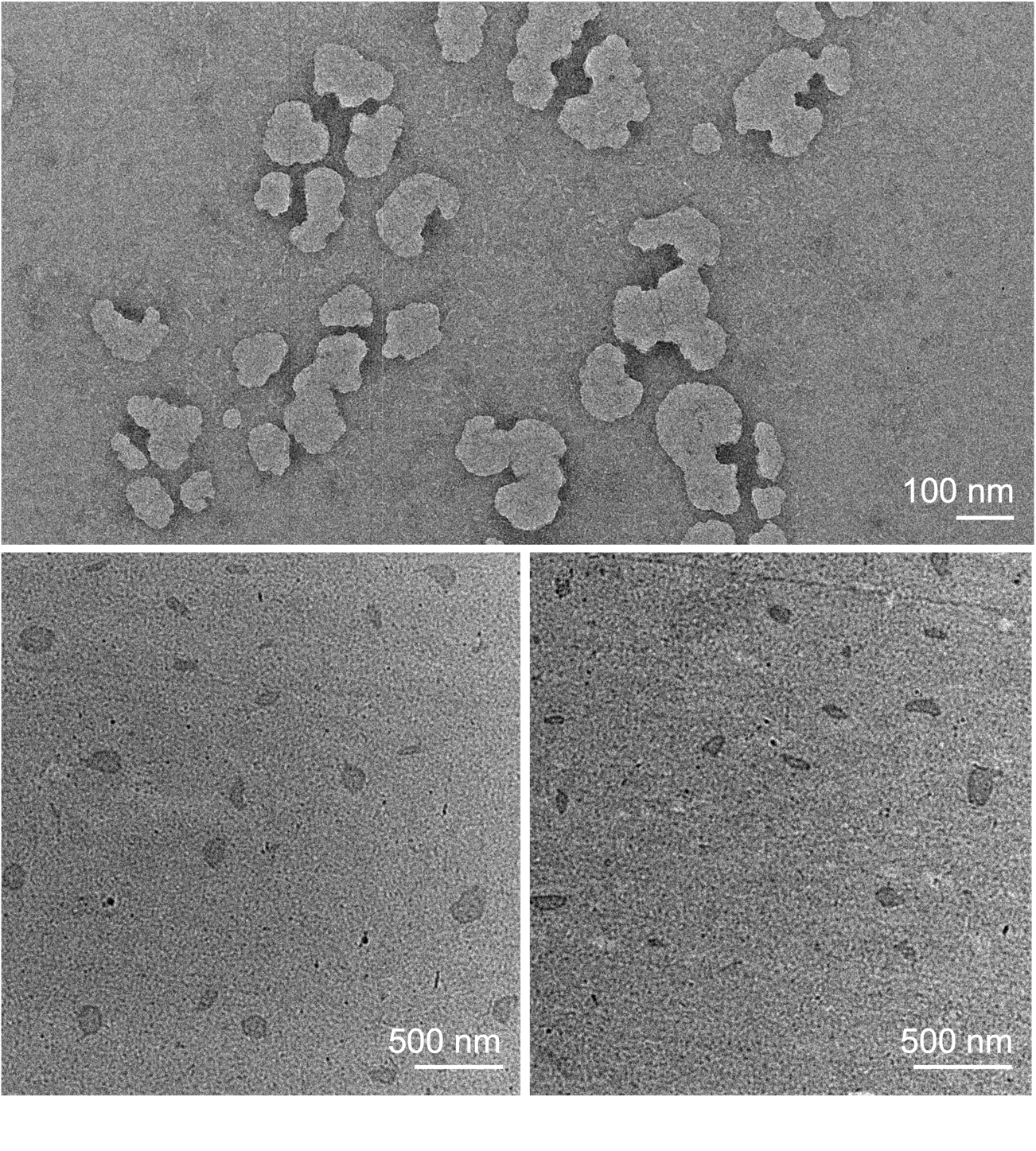
Figure S22.** TEM images of **AggIII** (*c* = 20 µM, 363 K K, H_2_O) deposited on carbon-coated copper grids. The top sample was stained with a 1% aqueous solution of uranyl acetate while the bottom ones were imaged without staining.

**Figure S23.** Experimental SAXS (*c* = 1 mM) profiles (circles) recorded at 293 K (a), 303 K (b) and 313 K (f) with corresponding fittings (solid lines) to the customized models. The obtained parameters are shown in Table S1. VT-SAXS measurements revealed a gradual increase in cylinder length upon heating, reaching a maximum at 313 K consistent with a thermally induced polymorphic transition from **AggI** to **AggII**. The slight deviation from the values observed by spectroscopy arises from differences in concentration.

**Table S1.** Sample details and fitting parameters of the experimental SAXS profiles of the aggregated species.

^a^ Recorded after cooling from 323 K.


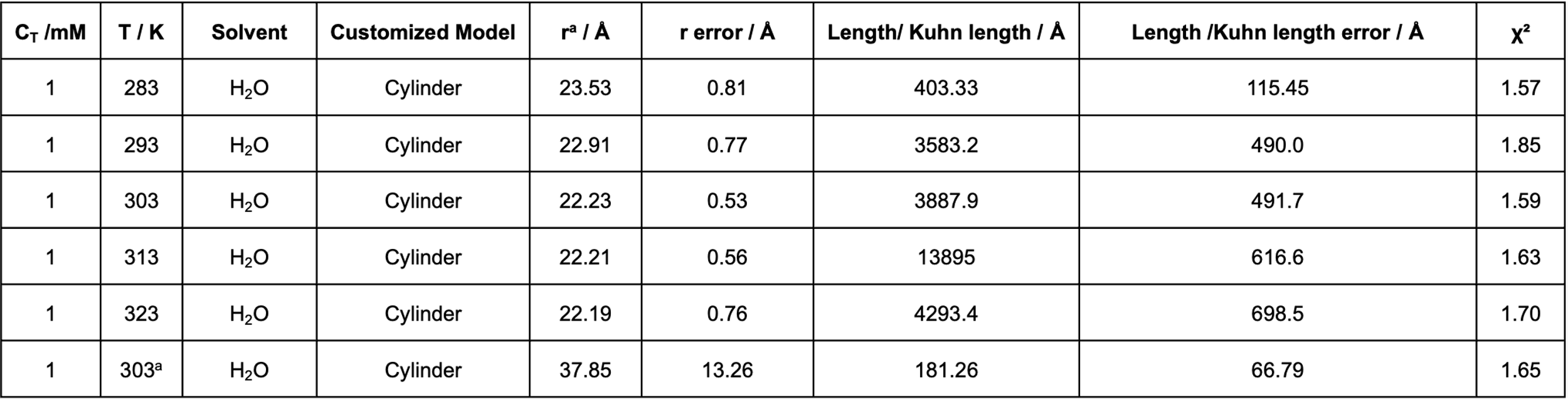


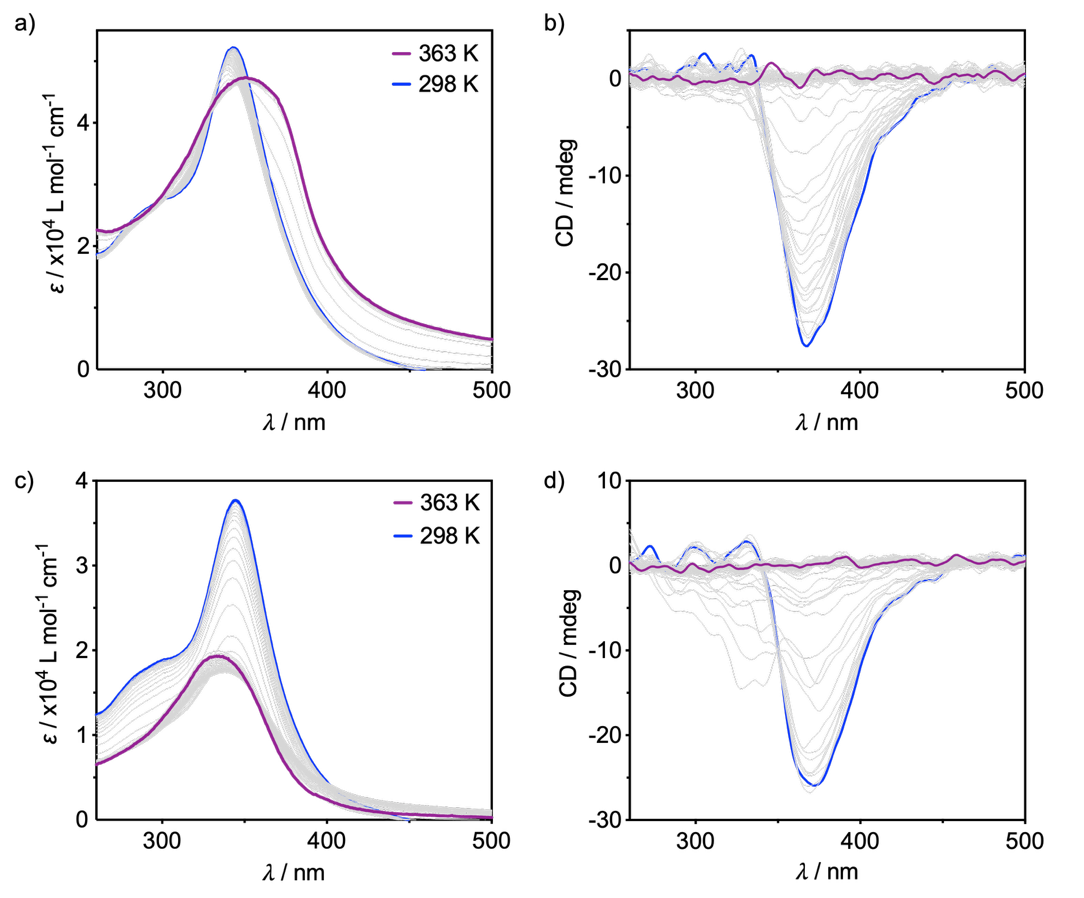
**Figure S24.** Cooling UV/Vis and CD studies of **1** in H_2_O:ACN 95:5 (v/v) (a, b) and H_2_O:ACN 90:10 (v/v) (c, d) (*c* = 20 µM).


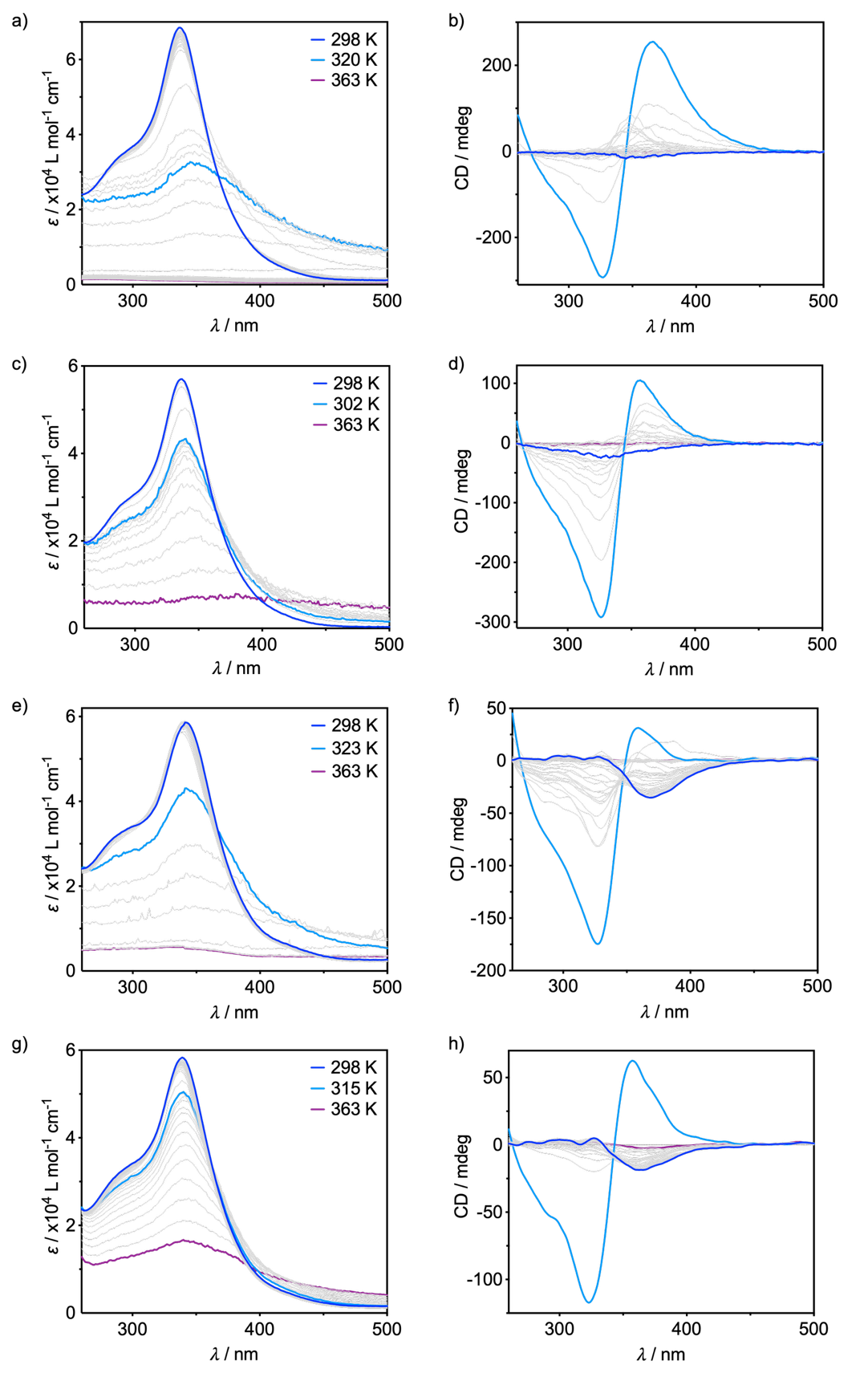
**Figure S25.** Heating UV/Vis and CD studies of **1** (*c* = 20 µM) in an aqueous solution of NaCl 0.5 M (a, b), NaCl 2.3 M (c, d), NaBr 0.5 M (e, f) and NaBr 2.3 M (g, h).


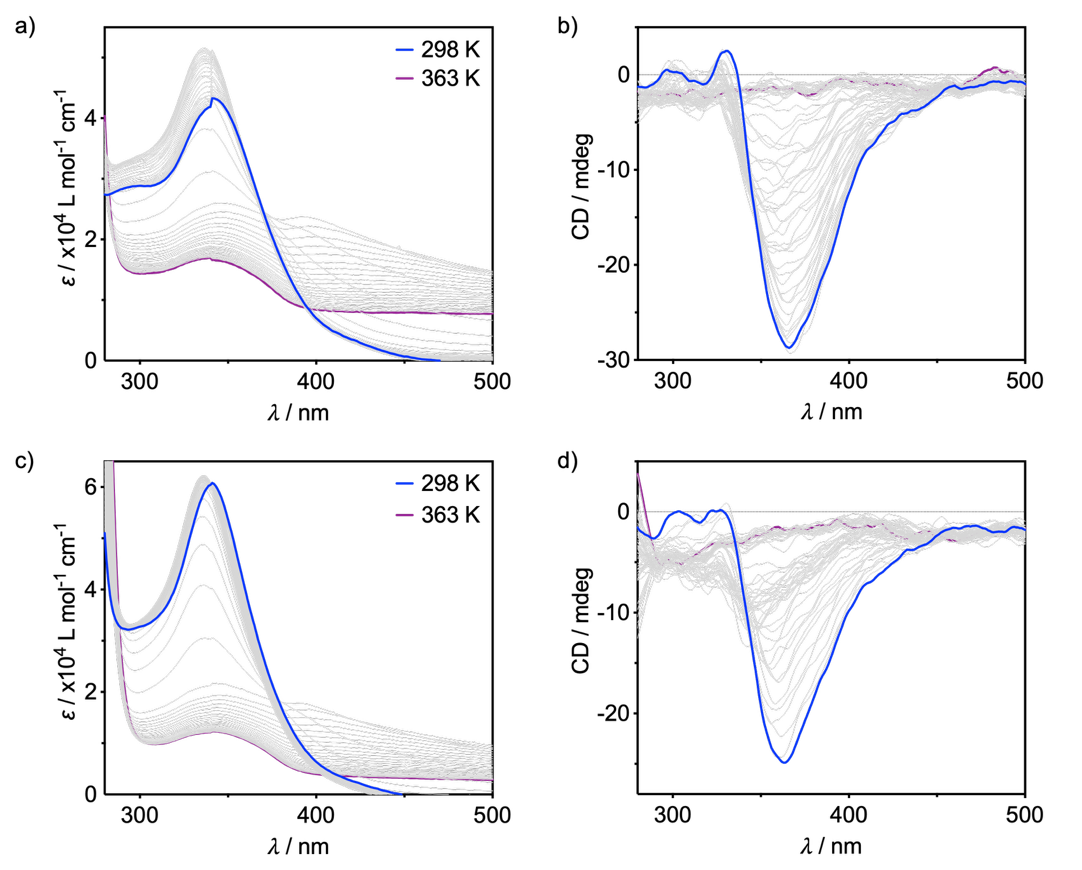


**Figure S26.** Heating UV/Vis and CD studies of **1** (*c* = 20 µM) in an aqueous solution of NaI 0.5 M (a, b) and NaI 2.3 M (c, d).


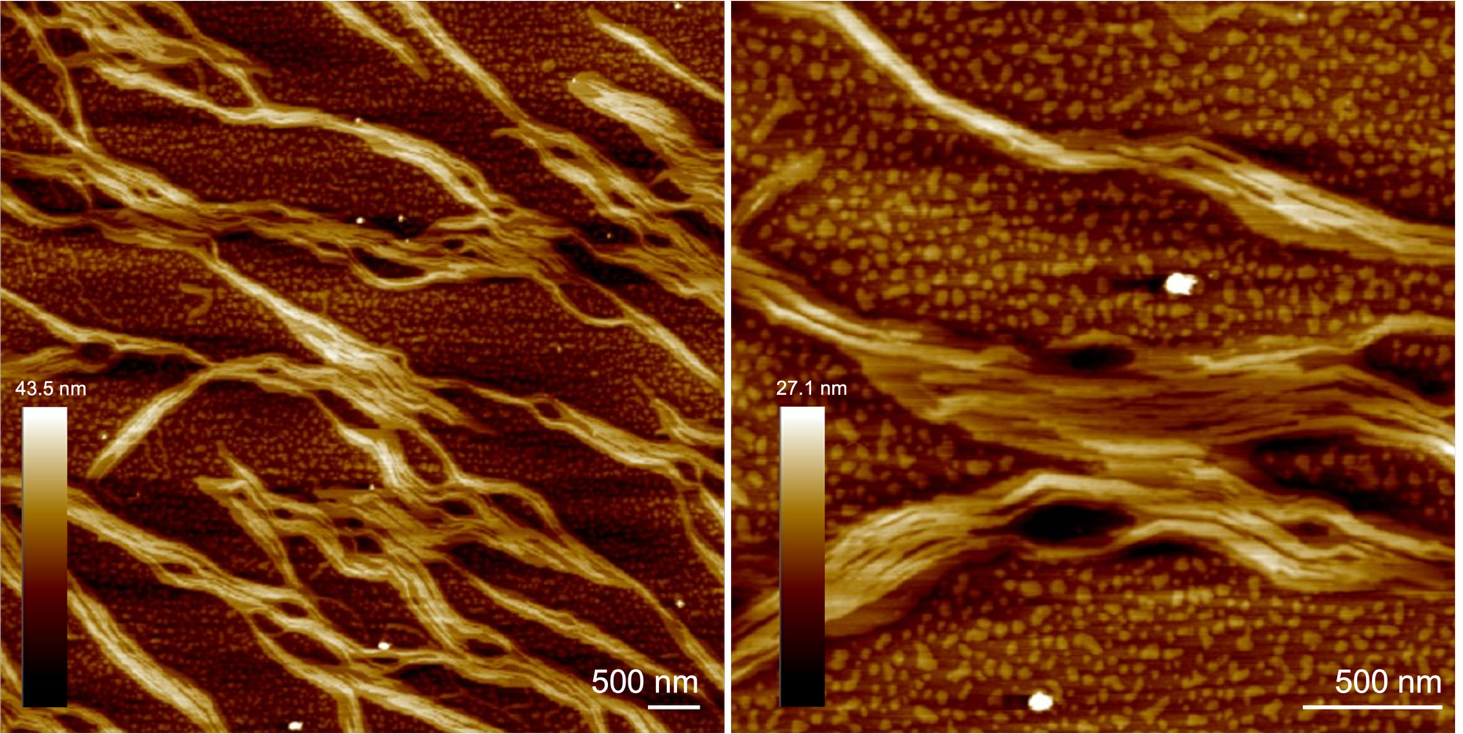

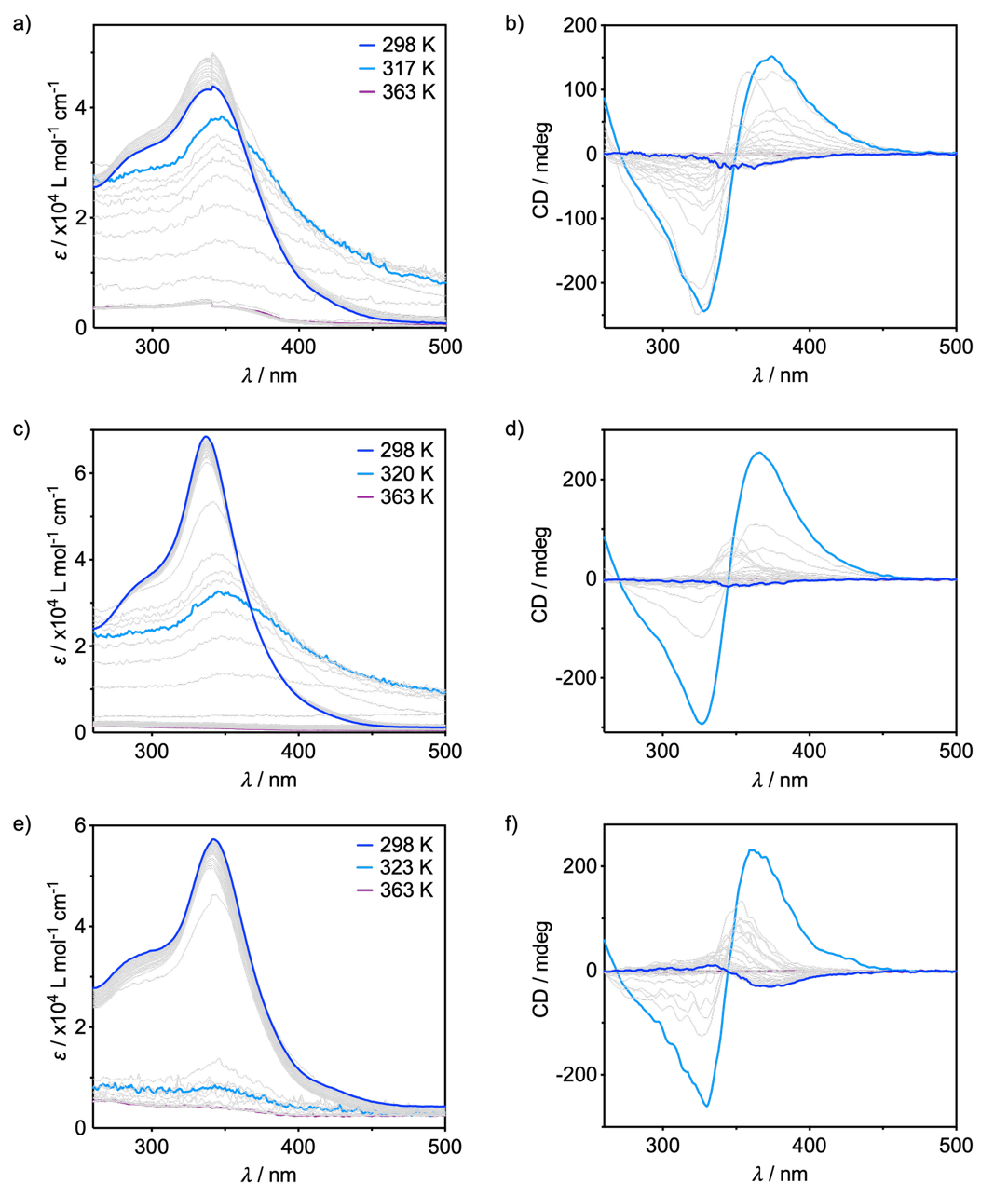
**Figure S27.** Heating UV/Vis and CD studies of **1** (*c* = 20 µM) in an aqueous solution of KCl 0.5 M (a, b), NaCl 0.5 M (c, d) and MgCl 0.5 M (e, f).

**Figure S28.** AFM images on mica obtained from a spin-coated solution of **AggII** (*c* = 20 µM, 1500 rpm, 298 K, H_2_O:ACN 85:15 (v/v)).


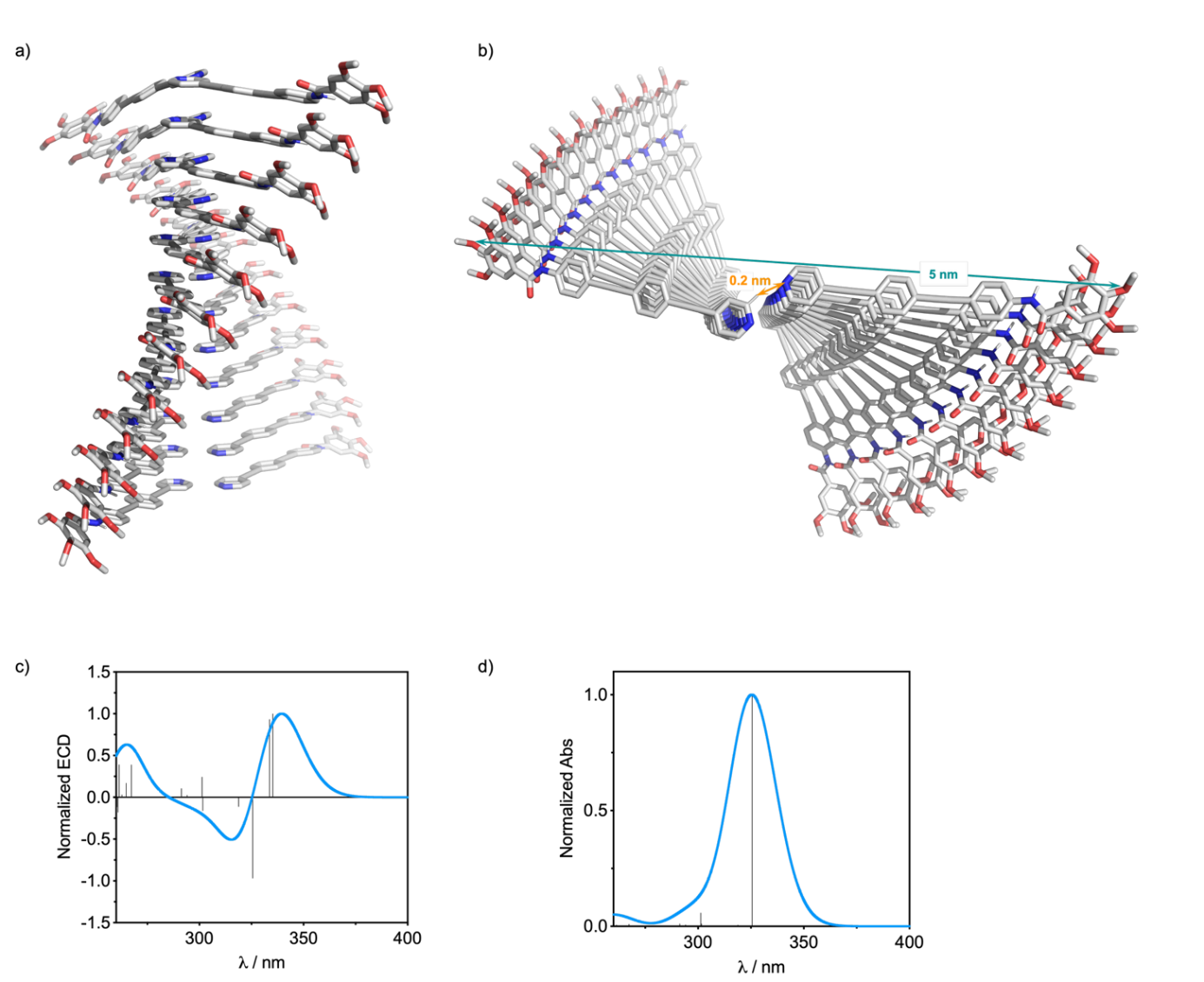
**Figure S29.** Top (a) and side (b) view of an optimized 24-mer assembly (12 monomer units on each stack) of **AggII**. The initial geometry was constructed based on experimental data extracted from microscopy studies (Figure 4). To reduce computational costs, the tetraethylene glycol chains were replaced by methyl groups and the optimized structure was obtained by using the GFN2-xTB method. The resulting structure reveals a (*P*)-oriented supramolecular assembly with a diameter of ca. 5 nm and a distance of 0.2 nm between the pyridine units. TD-DFT (rCAM-B3LYP/3-21G) theoretical CD (c) and UV/Vis (d) spectra of **AggII**, based on a tetramer extracted from the 24-mer stack.

# 3. References

[1] <https://www.sasview.org>

[2] C. Bannwarth, E. Caldeweyher, S. Ehlert, A. Hansen, P. Pracht, J. Seibert, S. Spicher, S. Grimme, Extended tight-binding quantum chemistry methods. *WIREs Comput. Mol. Sci.* **2020**, *11*, e1493.

[3] C. Bannwarth, S. Ehlert, S. Grimme, GFN2-xTB—An Accurate and Broadly Parametrized Self-Consistent Tight-Binding Quantum Chemical Method with Multipole Electrostatics and Density-Dependent Dispersion Contributions, *J. Chem. Theory Comput.* **2019**, *15*, 1652.

[4] M. D. Hanwell, D. E. Curtis, D. C. Lonie, T. Vandermeersch, E. Zurek, G. R. Hutchison, Avogadro: an Advanced Semantic Chemical Editor, Visualization, and Analysis Platform. *J. Cheminform*. **2012**, *4*,1-17.

[5] L. Borsdorf, L. Herkert, N. Bäumer, L. Rubert, B. Soberats, P. Korevaar, C. Bourque, C. Gatsogiannis, G. Fernández, Pathway-Controlled Aqueous Supramolecular Polymerization via Solvent-Dependent Chain Conformation Effects. *J. Am. Chem. Soc.* **2023**, *145*, 8882.

[6] O. Henze, W. J. Feast, F. Gardebien, P.Jonkheijm, R. Lazzaroni, P. Leclère, E. W. Meijer, A. P. H. J. Schenning, Chiral Amphiphilic Self-Assembled α, α′-Linked Quinque-, Sexi-, and Septithiophenes: Synthesis, Stability and Odd-Even Effects. *J. Am. Chem. Soc.* **2006**, *128*, 5923.

[7] A. Langenstroer, K. K. Kartha, Y. Dorca, J. Droste, V. Stepanenko, R. Q. Albuquerque, M. R. Hansen, L. Sánchez, G. Fernández, Unraveling Concomitant Packing Polymorphism in Metallosupramolecular Polymers*. J. Am. Chem. Soc.* **2019**, *141*, 5192.

[8] M. M. Smulders, M. M. L. Nieuwenhuizen, T. F. A. De Greef, P. Van der Schoot, A. P. H. J. Schenning, E. W. Meijer, How to Distinguish Isodesmic from Cooperative Supramolecular Polymerisation. *Chem. Eur. J.* **2010**, *16*, 362.
